# A case-control study of a combination of single nucleotide polymorphisms and clinical parameters to predict clinically relevant toxicity associated with fluoropyrimidine and platinum-based chemotherapy in gastric cancer

**DOI:** 10.1186/s12885-021-08745-0

**Published:** 2021-09-16

**Authors:** Miguel Cordova-Delgado, María Loreto Bravo, Elisa Cumsille, Charlotte N. Hill, Matías Muñoz-Medel, Mauricio P. Pinto, Ignacio N. Retamal, María A. Lavanderos, Juan Francisco Miquel, Maria Rodriguez-Fernandez, Yuwei Liao, Zhiguang Li, Alejandro H. Corvalán, Ricardo Armisén, Marcelo Garrido, Luis A. Quiñones, Gareth I. Owen

**Affiliations:** 1 Faculty of Chemical and Pharmaceutical Sciences, Universidad de Chile, 8380494 Santiago, Chile; 2 Department of Physiology, Faculty of Biological Sciences, Pontificia Universidad Católica de Chile, 8331150 Santiago, Chile; 3 Department of Hematology and Oncology, Faculty of Medicine, Pontificia Universidad Católica de Chile, 8330032 Santiago, Chile; 4 Millennium Institute on Immunology and Immunotherapy, 8331150 Santiago, Chile; 5 Faculty of Dentistry, Universidad de Los Andes, 7620001 Santiago, Chile; 6 Laboratory of Chemical Carcinogenesis and Pharmacogenetics, Department of Basic and Clinical Oncology, Faculty of Medicine, Universidad de Chile, 8380494 Santiago, Chile; 7 Latin American Network for Implementation and Validation of Clinical Pharmacogenomics Guidelines (RELIVAF-CYTED), Madrid, Spain; 8 Escuela de Química y Farmacia, Facultad de Ciencias Médicas, Universidad Bernardo O’Higgins, Santiago, Chile; 9 Department of Gastroenterology, Faculty of Medicine, Pontificia Universidad Católica de Chile, 8330032 Santiago, Chile; 10 Institute for Biological and Medical Engineering, Schools of Engineering, Medicine and Biological Sciences, Pontificia Universidad Católica de Chile, Santiago, Chile; 11 Central Laboratory, Yangjiang People’s Hospital, GuangDong Province, Yangjiang, China; 12 Center of Genome and Personalized Medicine, Institute of Cancer Stem Cell, Dalian Medical University, Dalian, China; 13 National Institute on Aging, National Institute of Health, Baltimore, USA; 14 Advanced Center for Chronic Diseases (ACCDiS), 8330034 Santiago, Chile; 15 Instituto de Ciencias e Innovación en Medicina, Facultad de Medicina, Clínica Alemana, Universidad del Desarrollo, 7590943 Santiago, Chile

**Keywords:** Predictive models, Single nucleotide polymorphism, Chemotherapy toxicity, Fluoropyrimidines, Platinums

## Abstract

**Background:**

Fluoropyrimidine plus platinum chemotherapy remains the standard first line treatment for gastric cancer (GC). Guidelines exist for the clinical interpretation of four DPYD genotypes related to severe fluoropyrimidine toxicity within European populations. However, the frequency of these single nucleotide polymorphisms (SNPs) in the Latin American population is low (< 0.7%). No guidelines have been development for platinum. Herein, we present association between clinical factors and common SNPs in the development of grade 3–4 toxicity.

**Methods:**

Retrospectively, 224 clinical records of GC patient were screened, of which 93 patients were incorporated into the study. Eleven SNPs with minor allelic frequency above 5% in *GSTP1*, *ERCC2*, *ERCC1*, *TP53*, *UMPS*, *SHMT1*, *MTHFR*, *ABCC2* and *DPYD* were assessed. Association between patient clinical characteristics and toxicity was estimated using logistic regression models and classification algorithms.

**Results:**

Reported grade ≤ 2 and 3–4 toxicities were 64.6% (61/93) and 34.4% (32/93) respectively. Selected *DPYD* SNPs were associated with higher toxicity (rs1801265; OR = 4.20; 95% CI = 1.70–10.95, *p* = 0.002), while others displayed a trend towards lower toxicity (rs1801159; OR = 0.45; 95% CI = 0.19–1.08; *p* = 0.071). Combination of paired SNPs demonstrated significant associations in *DPYD* (rs1801265), *UMPS* (rs1801019), *ABCC2* (rs717620) and *SHMT1* (rs1979277). Using multivariate logistic regression that combined age, sex, peri-operative chemotherapy, 5-FU regimen, the binary combination of the SNPs *DPYD* (rs1801265) + *ABCC2* (rs717620), and *DPYD* (rs1801159) displayed the best predictive performance. A nomogram was constructed to assess the risk of developing overall toxicity.

**Conclusion:**

Pending further validation, this model could predict chemotherapy associated toxicity and improve GC patient quality of life.

**Supplementary Information:**

The online version contains supplementary material available at 10.1186/s12885-021-08745-0.

## Introduction

Globally, gastric cancer (GC) is the sixth most common malignancy and the third leading cause of cancer death [1–4]. Current standard first-line treatment for GC patients consists of chemotherapy regimens that combine fluoropyrimidines and platinum compounds. Therapeutic responses and associated toxicity with these regimens can vary significantly among patients ranging from moderate to severe [5–7]. Indeed, gastrointestinal, hematological and neurological toxicities are commonly observed under these regimens, often leading to treatment discontinuation, a reduction in quality of life, and in some extreme cases to death [8, 9]. Hence, severe toxicity becomes an essential obstacle to treatment completion and predictive models of toxicity may improve patient quality of life by avoiding severe toxicity.

Previous studies have demonstrated that single nucleotide polymorphisms (SNPs) are associated with chemotherapy-associated toxicity [10–12]. This can be explained by gene variations that alter the enzymatic activity of key proteins affecting pharmacokinetic and pharmacodynamic processes [13, 14]. In this regard, platinum-based compounds can trigger cell arrest or apoptosis by forming Pt-DNA adducts [15]. Within our bodies, kidneys can excrete these compounds without undergoing biotransformation via B1/C2/G2 type ABC (ATP Binding Cassette) transporters [16, 17]. Within cells, metabolizing enzymes including GSTP1, GSTM1, NQO1 and SOD1 decrease intracellular levels of platinum compounds [18–21]. Intracellularly platinum compounds target the DNA forming DNA-Pt complexes. Damaged DNA is recognized by HMGB, an enzyme that coordinates DNA repair by nucleotide excision repair enzymes [19, 22]. On the other hand, 5-fluorouracil (5-FU) and its pro-drug capecitabine undergo a series of enzymatic transformations prior to exert their effects [23]. Although the precise mechanism is still unclear, 5-FU is known to inhibit thymidylate synthase (TYMS) suppressing the conversion of uracil into thymidylate, leading to the inhibition of DNA/RNA synthesis and eventually to cell death [24]. The metabolism of 5-FU occurs mainly in the liver, where DPYD metabolizes ~ 80% of the drug, producing 5,6 dihydroxy-5-FU (an inactive metabolite) [25]. It is widely documented that decreased DPYD activity is associated with severe toxicity [26–28]. Previous reports have also associated *TYMS* and *MTHFR* gene variations with 5-FU toxicity; however their clinical relevance is undetermined [29].

To date, the most reliable markers of fluoropyrimidine toxicity are DPYD*2A (rs3918290), DPYD-c.2846A > T (rs67376798), DPYD-Hap-B3 (rs56038477) and DPYD*13 (rs55886062). In fact, these are variants that have a well-documented association with severe toxicity associated with fluoropyrimidines, and there is a Clinical Pharmacogenetics Implementation Consortium (CPIC) guideline that recommends avoiding or reducing the dose of fluoropyrimidines if a patient carries any of these variants [30]. Unfortunately, given their low frequencies in the general population the use of these variants identifies only a small fraction of potentially at-risk patients. For example, their frequencies in the 1000-Genome (1000-G) project and gnomAD databases for American or Latino/Admixed American population are much lower than that of those published for European cohorts. According to these databases, the Latin-American frequencies of the risk allele for: DPYD * 2 is 0.1 and 0.2% (1000-G Project and gnomAD, respectively) yet an order of magnitude greater in frequency in a Finnish cohort (2.5%) [31, 32]. In Latin-America the DPYD-c.2846A > T frequency is 0.3 and 0.2% (1000-G Project and gnomAD, respectively); DPYD-Hap-B3 is 0.6 and 0.7% (1000-G Project and gnomAD), DPYD*13 is 0 and 0.007% (1000-G Project and gnomAD, respectively). Therefore, we hypothesize that common SNPs in the Latin American population can potentially explain the clinically relevant toxicity in patients with fluoropyrimidines and platinum-based treatment.

As previously mentioned, SNP variants have been previously correlated with chemotherapy toxicity [33–35]. Other factors such as chemotherapy scheme, dosage, sex, and age have also been implicated in the development and severity of toxicity [7, 36–38]. However, only a few studies have developed comprehensive models that incorporate genetic and non-genetic factors to predict toxicity [39–41]. Herein, we developed and tested several models based on clinical factors, treatment regimens and candidate-SNPs. Our best performance model was used to construct a nomogram.

## Materials and methods

### Patients and study design

A retrospective, observational and case/control study was carried out. A total of 224 gastric cancer patients diagnosed between April 2005 and March 2018 were registered at the UC-CHRISTUS Cancer Center in the Pontificia Universidad Católica de Chile (PUC). Previously, this group of patients has been clinically and molecularly characterized [42]. After applying the inclusion and exclusion criteria, a total of 93 CG patients were analyzed in this study. Eligibility criteria were: a) histologically confirmed GC, b) chemotherapy regimen based on fluoropyridines and/ or platinum compounds, c) adequate patient renal, hepatic and bone marrow function, determined by the treating physician at the time of starting chemotherapy, d) at least 2 cycles of chemotherapy, e) availability of biological sample for extraction of genetic material and f) adults (> 18 yr-old). Patients with neuropathies or hematological damage caused by other diseases were excluded. Clinical-pathological characteristics of patients included: age, sex, stage, ECOG, histological classification, treatment schemes used and co-comorbidities. The Ethics Committee at “PUC” approved this study (#16–046, April 21st, 2016) [4]. All participants signed an informed consent to participate in this study. A waiver of consent was granted to include deceased patients. All data were anonymized to protect patients’ privacy. This study strictly adhered to the Code of Ethics of the World Medical Association (Declaration of Helsinki, 1964).

### Toxicity graduation

Toxicities grades were determined following the National Cancer Institute Common Toxicity Criteria 4.0 (NCI-CTC 4.0). Data on anemia, neutropenia, febrile neutropenia, thrombocytopenia, nausea, vomiting, diarrhea, stomatitis, hand-foot syndrome, and peripheral neuropathy were collected. Then they were categorized into hematological, gastrointestinal, and neurological toxicity, and if they presented any of the above as “overall toxicity”. All association analysis evaluated grade 0–2 vs grade 3–4 for hematological, gastrointestinal, neurological and overall toxicity. Treatment schemes and supportive care are shown in Supplementary Data.

### SNP selection, DNA extraction and genotyping

A total of 11 SNPs were assessed, and a detailed description of this process is provided in Supplementary Data. Genotypic/allelic frequencies of analyzed SNPs are shown in Supplementary Table S8. Nucleic acids were extracted from paraffin-embedded tumor tissues using the “AllPrep DNA/RNA Mini Kit®” kit (Cat#AM1975, Thermo Fisher. DNA was quantified by “Qubit® dsDNA HS Assay” (Thermo Fisher). Candidate SNPs were genotyped by TaqMan® SNP Genotyping Assay technology on an Applied Biosystems® 7500 Fast Real-Time PCR System (Thermo Fisher). Samples were randomly reanalyzed for confirmation. TaqMan® probes are shown in Supplementary Table S9.

### Statistical analysis

#### Association analysis of SNPs

The association between SNPs and grade 3–4 toxicity was analyzed using univariate logistic regression models, reporting Odds Ratio (OR) values with 95% confidence interval (95% CI). These analyses were tested using 3 inheritance models; dominant, codominant and recessive, based on the parameters of AIC and BIC, the best inheritance model was chosen for each SNP [43]. To choose the SNP combinations in the first step, a multivariate logistic regression analysis was performed using the 11 SNPs. To reduce the number of combinations and avoid over-fitting, we applied the AIC-based “Stepwise algorithm”. On selected SNPs, binary combinations were performed and their association with grade 3–4 toxicity was established using their respective inheritance models.

#### Model building

We developed a total of 4 models for severe toxicity. Based on literature evidence or a *p*-value < 0.2, potentially relevant variables were included in each model. Multivariate logistic regression models were built incorporating variables that maximized Pseudo R^2^ (MacFadden); fit indicator of the variables to the model [41, 44]. For example, for overall toxicity Model 1 included: age, sex, peri-operative chemotherapy and scheme. Model 2 included the following SNPs: *ERCC2* (rs13181), *DPYD* (rs2297595), *DPYD* (rs1801159), DPYD (rs1801265) and *GSTP1* (rs1695). Model 3 included: age, sex, peri-operative chemotherapy, 5-FU containing scheme, *ERCC2* (rs13181), *DPYD* (rs2297595), *DPYD* (rs1801159), *DPYD* (rs1801265) and *GSTP1* (rs1695). Finally, model 4 included: age, sex, peri-operative chemotherapy, 5-FU containing scheme, paired SNPs *DPYD* (rs1801265) + *ABCC2* (rs717620), plus *DPYD* (rs1801159). For every type of toxicity, the selected variables are depicted in the corresponding table.

#### Model evaluation and nomogram construction

Obtained models were evaluated using classification algorithms [45, 46] including: Logistic Regression (LR); Support Vector Machine (SVM); Naïve Bayesian (NB); K-Nearest Neighbor (KNN); Artificial Neural Network (ANN); Random Forest (RF); Decision Tree (DT) (Details in Supplementary Data). Using as a basis the coefficients of the multivariate analysis of model 4 we constructed a nomogram using “rms” package [47]. In addition, for discriminatory capacity, 1000 bootstrap replications served as internal validation subsets to estimate the bias - corrected c - index calibration.

#### General statistical analysis

Continuous variables were compared using ANOVA. Kaplan-Meier method was used for survival analysis and log-rank tests for comparison. Significance was set at *p* < 0.05. According to the number of cases and controls (31 and 62 patients respectively), assuming a power of 80%, an α error of 5% and a frequency of common polymorphisms (i.e. *DPYD* rs1801265, *GSTP1* rs1695) of 30%, Odd Ratios could be detected with values of 3.9 and 0.1 (high and low). Association analysis of SNPs and overall toxicity were performed by SNPstat program [43]. Uni/multivariate logistic regression models were built using the “stats” and “DescTools” packages. Classification algorithms were constructed using the “caret” and “ROCR” packages. For survival analysis, the “survival” and “survminer” packages were used. All analysis were performed in R software v3.5.1 (The R Foundation, Vienna, Austria). Full datasets used in this study can be found in Supplementary Data File 1.

## Results

### SNPs selection

SNPs were selected based on: (1) scientific evidence regarding the SNPs/toxicity relationship, using the PharmGKB database [48]; (2) allelic and genotypic frequency of the SNPs in the American population [32]; (3) relationship of the SNPs with the toxicity collected in our patients literature-based criteria; (4) functional impact of SNPs at the protein level according to PolyPhen [49] and SIFT [50]. Briefly, in a first approximation 27 SNPs in 11 genes and 7 SNPs in 6 genes for fluoropyrimidines and platinums were reviewed, respectively. Then, based on a score system for fluoropyrimidines, 14 SNPs in 7 different genes were candidates, while for platinums, 4 SNPs in 4 different genes were candidates. Finally, eleven SNPs with an allelic frequency greater than 5% were genotyped (Table 1). A detailed description of this process is provided in Supplementary Data.

**Table 1:** Brief description of the SNPs analyzed in this study

Gen	SNP ID	AA Change	SNPs effect	Genotype associated to toxicity	Allelic frequency American population (n)^a^	Toxicity
*GSTP1*	rs1695, A > G	I105V	Protein alteration	AA and AG	Allele A: 0.52	↑ risk of hematological toxicity
*ERCC2*	rs13181, T > G	K751Q	Protein alteration	GG and GT	Allele G: 0.21	↑ risk of hematological toxicity
*TP53*	rs1042522, C > G	P72R	Protein alteration	CG and GG	Allele G: 0.32	↑ risk of hematological toxicity
*UMPS*	rs1801019, G > C	G213A	Protein alteration	CC	Allele C: 0.26	↑ risk of gastrointestinal toxicity
*SHMT1*	rs1979277, G > A	L474F	Protein alteration	AA	Allele A: 0.27	↑ risk of toxicity
*MTHFR*	rs1801131, T > G	E429A	Protein alteration	TT	Allele T: 0.15	↑ risk of toxicity
*ABCC2*	rs717620, C > T	–	Change in 5`-UTR	TT	Allele T: 0.17	↑ risk of neurotoxicity
*ERCC1*	rs11615, A > G	N118=	Synonymous change	AA	Allele A: 0.39	↑ risk of hematological toxicity
*DPYD*	rs2297595, T > C	M166V	Protein alteration	CC and CT	Allele C: 0.06	↑ risk of severe toxicity
*DPYD*	rs1801159, T > C	I543V	Protein alteration	CC and CT	Allele C: 0.27	↑ risk of severe toxicity
*DPYD*	rs1801265, A > G	C29R	Protein alteration	GG and AG	Allele G: 0.22	↑ risk of severe toxicity

^a^ Frequencies of genotypes associated to toxicity in American population according 1000-G project

### General characterization of patients

Main clinicopathological characteristics of the patients are summarized in Supplementary Table S1. Briefly, patients were predominantly male (62.4%) and advanced stage III / IV (71.0%). Most tumors were located in the stomach corpus (31.2%). Histologically, 32.3% were diffuse-type by Lauren and 77.3% were gastric adenocarcinomas. Survival curves are shown in Supplementary Fig. S1. Median overall survival (OS) for the entire group was 29 months (Supplementary Fig. S1A). Males displayed better OS versus females, however these differences did not reach statistical significance (Log Rank *p* = 0.45, Supplementary Fig. S1B). As expected, stage had a significant impact on OS. Advanced stage patients showed lower median OS (30 or 13 months for stages III or IV, respectively) versus early stage (62 months in stage II, not reached for stage I) (Supplementary Fig. S1C, Log Rank *p* < 0.0001). Supplementary Table S2 summarizes treatment characteristics. FOLFOX was the most frequently used schema (49.5%) followed by CAPEOX and CF (18.3 and 14.0%, respectively). Adverse reactions are summarized in Supplementary Table S3. Data were grouped according to type of toxicity. Peripheral neuropathy was the most common grade 1 toxicity (34.1%). Nausea was the most predominant grade 2 toxicity (31.5%). Among grade 3 toxicities, neutropenia was dominant (22.58%) followed by diarrhea (20%). Finally, we registered a total of 5 patients with grade 4 events among these 3 out of 5 corresponded to neuropathy (60%). No toxicity-related deaths were registered. Clinically relevant toxicities (grade ≥ 3) were more frequently associated to digestive problems such as diarrhea and stomatitis, with a total of 19 registered events. Among hematological toxicities, a total of 17 grade ≥ 3 events, principally neutropenia or febrile neutropenia, were registered (Supplementary Table S3).

### Association between non-genetic factors and overall toxicity

A 34.4% of patients (32/93) displayed grade ≥ 3 overall toxicity. Non-genetic factors and ≥ 3 grade overall toxicity associations are shown in Tables 2 and 3. No significant associations were found between clinicopathological variables and serious adverse reactions. In agreement with previous reports, elderly patients have an OR = 1.83 (95% CI = 0.77–4.49), female patients had an OR = 1.40 (95% CI = 0.57–3, 40) and ECOG = 2 patients displayed an OR = 4.00 (95% CI = 0.35–90.53). However, none of these associations reached statistical significance (Table 2). Regarding treatment associated factors (Table 3), patients that received peri-operative chemotherapy regimens had higher grade 3–4 toxicity rates compared to adjuvant treatment (OR = 2.71, 95% CI = 0.91–8.62, *p* = 0.07, peri vs. adj). Given the heterogeneity of the used schemes in our study, we classified them according to drug contents; 5-FU-containing schemes were associated to higher toxicity OR = 2.26 (95% CI: 0.79–7.48, *p* = 0.12). In contrast, capecitabine-containing schemes displayed lower toxicity OR = 0.52 (95% CI = 0.15–1.50, *p* = 0.23). However, none of these associations reached statistical significance.

**Table 2:** Univariate logistic regression analysis for the association between clinicopathological variables and overall toxicity grade ≥ 3

Characteristic	Control (***n*** = 61) n (%)	Case (***n*** = 32) n (%)	OR 95% CI	p-value
**Age median (years)**				
< 59	32 (52.5)	12 (37.5)	Ref.	0.16
≥ 59	28 (45.9)	20 (62.5)	1.83 (0.77–4.49)	
**Sex**				
Male	41 (67.2)	19 (59.4)	Ref.	0.45
Female	20 (32.8)	13 (40.6)	1.40 (0.57–3.40)	
**Stage**				
I-II	20 (32.8)	7 (21.9)	Ref.	0.26
III-IV	41 (67.2)	25 (78.1)	1.74 (0.67–4.97)	
**ECOG**				
0	28 (45.9)	14 (43.8)	Ref.	0.44
1	26 (42.6)	11 (34.4)	0.84 (0.32–2.19)	
2	1 (1.6)	2 (6.3)	4.0 (0.35–90.53)	
NA	6 (9.8)	5 (15.6)		
**Lauren histotype**				
Diffuse	20 (32.8)	10 (31.3)	Ref.	0.98
Intestinal	17 (27.9)	9 (28.1)	1.05 (0.34–3.22)	
Mixed	7 (11.5)	4 (12.5)	1.14 (0.25–4.77)	
NA	17 (27.9)	9 (28.1)		
**Signet-ring cells**				
No	35 (57.4)	19 (59.4)	Ref.	0.85
Yes	26 (42.6)	13 (40.6)	0.92 (0.38–2.18)	
**Comorbidities**				
Chronic heart disease	5 (8.2)	2 (6.3)	0.74 (0.10–3.69)	0.73
Chronic liver disease	1 (1.1)	1 (3.3)	1.93 (0.07–50.06)	0.64
Thromboembolic event	1 (1.1)	1 (3.3)	1.93 (0.07–50.06)	0.64
Diabetes	9 (14.8)	2 (6.3)	1.10 (0.26–3.97)	0.88

*NA* not available, *ECOG* Eastern Cooperative Oncology Group

Significance: *P* < 0.05

**Table 3:** Univariate logistic regression analysis for association between treatment variables and overall grade ≥ 3 toxicity

Characteristic	Control (n = 61) n (%)	Case (n = 32) n (%)	OR [95% CI]	p-value
**Chemotherapy regimen**				
Adjuvant	19 (31.1)	7 (21.9)	Ref.	0.21
Peri-operative	16 (26.2)	16 (50)	2.71 (0.91–8.62)	
Palliative	22 (36.1)	8 (25)	0.98 (0.29–3.30)	
CMT + RDT Adjuvant	3 (4.9)	1 (3.1)	0.90 (0.04–8.52)	
CMT + RDT Peri-operative	1 (1.6)	0	NA	
**Chemotherapy scheme**				
FOLFOX	29 (47.5)	17 (53.1)	Ref.	0.07
CAPEOX	12 (19.7)	5 (15.6)	0.71 (0.19–1.05)	
CF	7 (11.5)	6 (18.8)	1.46 (0.40–5.12)	
DCFm	5 (8.2)	0	NA	
ECF	0	2 (6.3)	NA	
EOX	2 (3.3)	0	NA	
Capecitabine	2 (3.3)	0	NA	
FLOT	0	1 (3.1)	NA	
RDT + 5-FU/Leu	2 (3.3)	1 (3.1)	0.85 (0.03–9.55)	
RDT + CAPEOX	1 (1.6)	0	NA	
RDT + Cis/Cape	1 (1.6)	0	NA	
**Previously treated**				
No	53 (86.9)	30 (93.7)	Ref.	
Yes	8 (13.1)	2 (6.3)	0.44 (0.06–1.90)	0.29
**Scheme contains**				
RDT	4 (6.6)	1 (3.1)	0.45 (0.02–3.27)	0.46
5-FU	43 (70.5)	27 (84.4)	2.26 (0.79–7.48)	0.12
Capecitabine	16 (26.2)	5 (15.6)	0.52 (0.15–1.50)	0.23
Oxaliplatin	44 (72.1)	23 (71.9)	0.81 (0.30–2.21)	0.67
Cisplatin	13 (21.3)	8 (25.0)	1.23 (0.43–3.33)	0.68
Docetaxel	5 (8.2)	1 (3.1)	0.36 (0.01–2.37)	0.31

*CMT. chemotherapy; RDT. Radiotherapy; FOLFOX. 5-fluorouracil + oxaliplatin + leucovorin; CAPEOX. capecitabine + oxaliplatin;* CF. *cisplatin + 5-fluorouracil; DCFm. docetaxel + cisplatin + 5-fluorouracil; ECF. etoposide + cisplatin + 5-fluorouracilo; FLOT. 5-fluorouracil + oxaliplatin + docetaxel + leucovorin; 5FU. 5-fluorouracil; Leu. leucovorin; Cis. cisplatin; Cape. capecitabine; NA, not applicable; Ref. Reference*

*Significance: P < 0.05*

### Genetic variants associated with overall toxicity

Binary associations between overall toxicity and SNPs were assessed using three inheritance models (codominant, dominant and recessive) and are summarized in Table 4. Using a dominant model, the AG/GG genotypes of SNPs in the *DPYD* (rs1801159) were associated with higher toxicity; OR = 4.20 (95% CI = 1.70–10.95, *p* = 0.002). Also, we found a borderline association between lower toxicity and *DPYD* (rs1801159) with an OR = 0.45 (95% CI = 0.19–1.08; *p* = 0.071). Potential associations in *DPYD* (rs2297595), *ERCC2* (rs13181) and *GSTP1* (rs1695) SNPs were also analyzed. However, no significant association was found by univariate analysis.

**Table 4:** SNPs and overall grade ≥ 3 toxicity associations in GC patients treated with platinum/fluoropyrimidines-based chemotherapy

Gen SNP ID	Model	Genotypes	Control (n = 61) n (%)	Case (n = 32) n (%)	OR [95% CI]	p-value
***ERCC2***	Dom	T/T	39 (63.9%)	16 (50%)	Ref.	0.20
rs13181 T > G		T/G-G/G	22 (36.1%)	16 (50%)	1.77 (0.74–4.22)	
***DPYD***	–	T/T	57 (95%)	28 (87.5%)	Ref	
rs2297595 T > C		C/T	3 (5%)	4 (12.5%)	2.71 (0.57–12.97)	0.21
***DPYD***	Dom	T/T	22 (36.7%)	18 (56.2%)	Ref	
rs1801159 T > C		C/T-C/C	38 (63.3%)	14 (43.8%)	0.45 (0.19–1.08)	0.071
***DPYD***	Dom	A/A	40 (66.7%)	10 (32.3%)	Ref	
rs1801265 A > G		A/G-G/G	20 (33.3%)	21 (67.7%)	**4.20 (1.70–10.95)**	**0.002**
***GSTP1***	Dom	A/A	22 (36.1%)	14 (43.8%)		
rs1695 A > G		A/G-G/G	39 (63.9%)	18 (56.3%)	0.72 (0.30–1.74)	0.47

*Dom*: Dominant inheritance model

Significance: *P* < 0.05

### Combination of genetic variants associated with overall toxicity

Next, we performed a multivariate logistic regression analysis incorporating the 11 SNPs to establish potential associations between combined SNPs and overall toxicity. We applied a “Stepwise algorithm” based on Akaike information criterion (AIC) [51] to reduce the number of combinations and avoid overfitting. Based on this we selected, 5 SNPs and their respective inheritance models to test binary combinations between SNPs (Table 5). In respect to SNP combinations, those patients that carry the AG/GG + GG/GC genotype combination in *DPYD* (rs1801265) and *UMPS* (rs1801019) are at a higher risk to develop toxicity; OR = 4.22 (95% CI = 1.66–11.40, *p* = 0.0031) versus AA + GG / GC genotype patients. Furthermore, the *DPYD* (rs1801265)/*SHMT1* (rs1979277) combination displayed a borderline association with grade 3–4 overall toxicity. In this case, the AG/GG + GG genotype had a higher toxicity versus AA + GG; OR = 3.25 (95% CI = 0.99–11.39, *p* = 0.055). Finally, the *DPYD* (rs1801265)/*ABCC2* (rs717620) combination showed a strong association with grade 3–4 toxicity. Thus, patients carrying the AG/GG + CT/TT genotype had a higher probability of developing toxicity; OR = 11.25 (95% CI = 1.25–245.45, *p* = 0.047) against the AA + CC genotype.

**Table 5:** Combination of SNPs with overall grade ≥ 3 toxicity associations in gastric cancer patients treated with platinum/fluoropyridines -based chemotherapy

SNPs combination	Control (n = 61) n (%)	Case (n = 32) n (%)	OR [95% CI]	p-value
*DPYD* dom (rs1801265) **+** ***UMPS*** **rec (rs1801019)**				
AA + **GG/GC**	38 (63.3)	9 (29.0)	ref.	–
AA + **CC**	2 (3.3)	1 (3.2)	2.11 (0.09–24.53)	0.55
AG/GG + **GG/GC**	20 (33.3)	20 (64.5)	**4.22 (1.66–11.40)**	**0.0031**
AG/GG + **CC**	0 (0)	1 (3.2)	NA	NA
*DPYD* dom (rs1801265) + ***SHMT1*** **dom (rs1979277)**				
AA + **GG**	19 (32.2)	7 (22.6)	ref.	–
AA + **GA/AA**	20 (33.9)	3 (9.7)	0.40 (0.07–1.69)	0.23
AG/GG + **GG**	10 (16.9)	12 (38.7)	**3.25 (0.99–11.39)**	**0.055**
AG/GG + **GA/AA**	10 (16.9)	9 (29.0)	2.44 (0.70–8.84)	0.16
*DPYD* dom (rs1801265) + ***ABCC2*** **dom (rs717620)**				
AA + **CC**	30 (50.0)	8 (25.8)	ref.	–
AA + **CT/TT**	10 (16.7)	2 (6.5)	0.75 (0.10–3.64)	0.74
AG/GG + **CC**	19 (31.7)	18 (58.1)	**3.55 (1.32–10.20)**	**0.014**
AG/GG + **CT/TT**	1 (1.7)	3 (9.7)	**11.25 (1.25–245.45)**	**0.047**

*Dom* Dominant inheritance model, *Rec* Recessive inheritance model

Significance: *P* < 0.05

### Multivariate analysis for the development of prediction models

Next, we sought to determine if the addition of multiple genetic factors and clinical/treatment information delivered a better prediction. We developed four models with different variables using the multivariate logistic regression. Model 1 was restricted to clinical/treatment variables. Model 2 included only SNPs. Model 3 incorporated a combination of clinical and treatment variables plus SNPs. Finally, model 4 was a mixture of clinical/treatment variables and paired SNPs (see methods). Selection of variables for each model was based on maximum Pseudo R^2^. Variables, ORs and Pseudo R^2^ for each model are shown in Table 6. Interestingly, the addition of clinical variables and genotypes improved model performance. For model 1 Pseudo R^2^ values were 0.073 and 0.15, respectively. In contrast, for combined models (model 3 and 4) values were 0.21 and 0.21, respectively. Furthermore, when we analyzed the SNPs in *DPYD* (rs1801159) in model 2 OR increased from 3.74 to 4.55 (*p* = 0.004) after adding clinical variables (model 3). Interestingly, sex has been reported to be a determining factor in the effects of polymorphisms on *DPYD* [7]. In line with the literature, the association with grade 3–4 toxicity of AG/GG genotypes in relation to AA had an OR = 7.78 (95% CI = 2.31–31.79, *p* = 0.001) or 1.85 (95% CI = 0.43–8.26, *p* = 0.40) for male or female patients, respectively (see Supplementary Table S4). On the other hand, in model 4 the *DPYD* (rs1801265) + *ABCC2* (rs717620) pair displayed a strong association, in this case the combination AG/GG + CT/TT genotype showed an OR = 18.00 (95% CI = 1.66–439, *p* = 0.027) versus AA + CT/TT.

**Table 6:** Models for overall grade ≥ 3 toxicity in gastric cancer patients treated with platinum/fluoropyridines -based chemotherapy using multivariate analysis

	Model 1		Model 2		Model 3		Model 4	
Characteristics	OR [95% CI]	p-value	OR [95% CI]	p-value	OR [95% CI]	p-value	OR [95% CI]	p-value
Age (above median)	1.71 (0.67–4.48)	0.25	–	–	2.33 (0.81–7.15)	0.12	2.24 (0.77–6.96)	0.14
Sex (Female)	1.83 (0.68–5.05)	0.23	–	–	2.12 (0.68–7.01)	0.19	2.18 (0.70–7.29)	0.18
CMT peri-op (Yes)	2.66 (1.00–7.35)	0.05	–	–	2.26 (0.73–7.28)	0.15	3.01 (0.94–10.31)	0.06
5-FU based (Yes)	1.73 (0.55–6.10)	0.35	–	–	2.03 (0.58–8.06)	0.28	1.65 (0.46–6.50)	0.44
**SNPs**								
*ERCC2* (dom. TG/GG)	–	–	1.56 (0.57–4.31)	0.37	1.24 (0.41–3.63)	0.68	–	–
*DPYD* (CT) (rs2297595)	–	–	1.56 (0.26–9.95)	0.61	1.88 (0.25–14.77)	0.52	–	–
*DPYD* (dom. TT) (rs1801159)	–	–	2.32 (0.89–6.24)	0.08	2.53 (0.91–7.44)	0.07	–	–
*DPYD* (dom. AG/GG) (rs1801265)	–	–	3.74 (1.46–10.08)	0.006	4.55 (1.64–13.79)	0.004	–	–
*GSTP1* (dom. AG/GG) (rs1695)	–	–	0.71 (0.26–1.90)	0.50	0.59 (0.19–1.74)	0.34	–	–
**SNPs combination**								
*DPYD* dom. + *ABCC2* dom. (rs1801265 + rs717620)	–	–	–	–			–	–
AA + CT/TT	–	–	–	–			1.45 (0.18–8.68)	0.68
AG/GG + CC	–	–	–	–			4.33 (1.43–14.68)	0.012
AG/GG + CT/TT	–	–	–	–			18.00 (1.66–439)	0.027
*DPYD* (dom. TT) (rs1801159)	–	–	–	–			2.37 (0.87–6.75)	0.094
	**Model 1**		**Model 2**		**Model 3**		**Model 4**	
**Pseudo R**^**2**^	0.07		0.15		0.21		0.21	

*CMT* Chemotherapy, *Dom* Dominant inheritance model

Significance: *P* < 0.05

### Evaluation of toxicity models

**Fig. 1:**
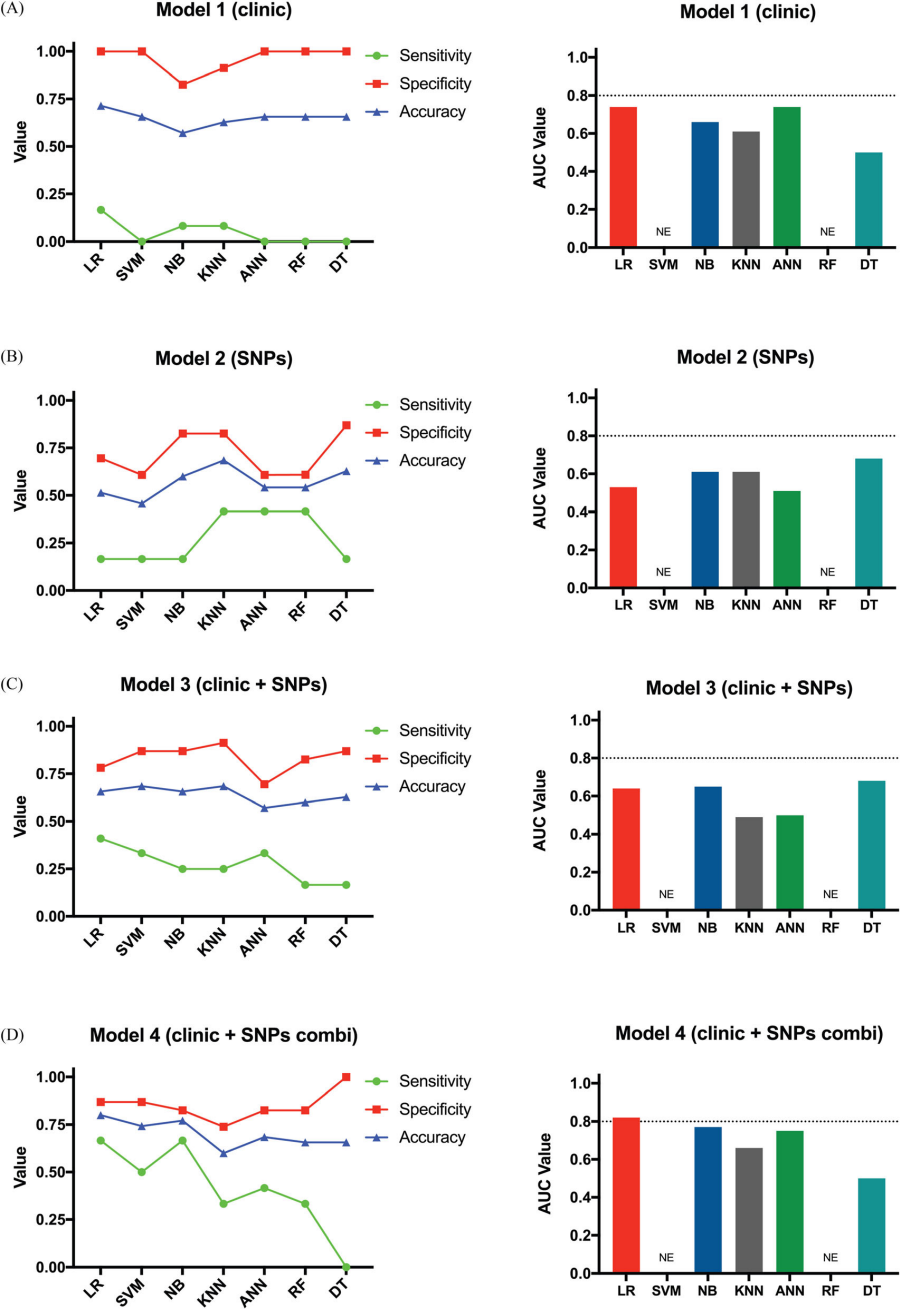
Performance of platinum/fluoropyrimidines-based chemotherapy toxicity prediction models using different classification algorithms. The sensitivity (green line), specificity (red line), accuracy (blue line) and AUCs of ROC curve of models (bar figure) were shown. Overall toxicity prediction model 1 (**A**), model 2 (**B**), model 3 (**C**) and model 4 (**D**). LR. logistic regression; SVM. support vector machine; NB. naïve Bayesian; KNN. k-nearest neighbor; ANN. artificial neural network; RF. random forest; DT. decision tree. N.E; not evaluated

To assess the predictive power of our models we employed a variety of classification algorithms. Figure 1 shows the sensitivity, specificity, accuracy and AUC of each model. For example, model 1 had a high specificity (range = 0.82–1.0), but low sensitivity (range = 0–0.17), in most tested algorithms. Accuracy reached a maximum value of 0.71 using the RL method and an AUC of 0.74 in the RL and ANN classification algorithms (Fig. 1A). Comparing different algorithms for model 2, were found a promising specificity (range = 0.61–0.87) but low sensitivity (range = 0.17–0.42). In this model maximum accuracy (0.69) was achieved with the KNN method, and the most favorable AUC was 0.68 with the DT method (Fig. 1B). Model 3 showed a relatively high specificity (range = 0.70–0.91) and a moderately low sensitivity (range = 0.17–0.41), reaching its maximum value with the RL method. On the other hand, the maximum accuracy was 0.69 with the SVM and KNN methods, and a maximum AUC of 0.68 achieved with the DT method (Fig. 1C). Model 4 showed a relatively high specificity (range = 0.74–1.0), with the sensitivity ranging between 0 and 0.66 among the classification algorithms. Maximum accuracy was achieved with the RL method (0.80); AUC was 0.82 achieved with the same classification algorithm (Fig. 1D). In summary, our data suggest model 4 was the best predictor of grade 3–4 toxicity (using the RL method). This supports the notion that combined models provide better predictive power versus individual variable models.

### Multivariate analysis for type of toxicity

In line with the methodology employed for general toxicity, multivariate analysis was performed on clinical and genetic factors in the development of severe toxicity for independent hematological, gastrointestinal or neurological toxicities. For hematological toxicity, when the clinical/treatment variables were integrated with SNPs, a better fit of the logistic regression model was achieved. For example, for model 1 and model 2, the Pseudo R^2^ returned values of 0.07 and 0.14, respectively, while for the variable’s integration models vary between 0.23 and 0.29 (Supplementary Table S5). Analysis of model 4 (Supplementary Table S5) shows that the variables of sex and the binary combination between the SNPs in ERCC1 (rs11615) and GSTP1 (rs1695) plus the SNP in DPYD (rs1801265) were either associated significantly or at least showed a strong tendency with hematological toxicity. For gastrointestinal toxicity, we observed that the clinical variables have a low influence on the presentation of this toxicity (Pseudo R^2^ = 0.04). An improved fit is observed in upon the examination of the 3 SNPs (Pseudo R^2^ value of 0.15; model 2). Upon combination of the clinical/treatment variables and genotypes, there is a modest increase in the Pseudo R^2^ value to 0.20 and 0.18, for models 3 and 4, respectively (Supplementary Table S6). Applying the same analysis for neurological toxicity, only 3 models could be developed, where model 1, which includes only clinical factors / treatment, had a better fit (Pseudo R^2^ = 0.15) than model 2 (Pseudo R^2^ = 0.15) which included only the SNPs in TP53 (rs1042522). Interestingly, in neurological toxicity, model 3, which includes both types of variables, had a Pseudo R^2^ value of 0.19, the highest value among these models (Supplementary Table S7). Taken together, these results suggest that it is the combined models that provide higher Pseudo R^2^ values. However, given the reduced number of variables (low number of SNP incidence) when individual toxicities are analyzed the associations may be relatively imprecise (reflected in the confidence intervals) and thus must be interpreted with caution until a larger cohort is studied.

### Nomogram for predicting general toxicity

**Fig. 2:**
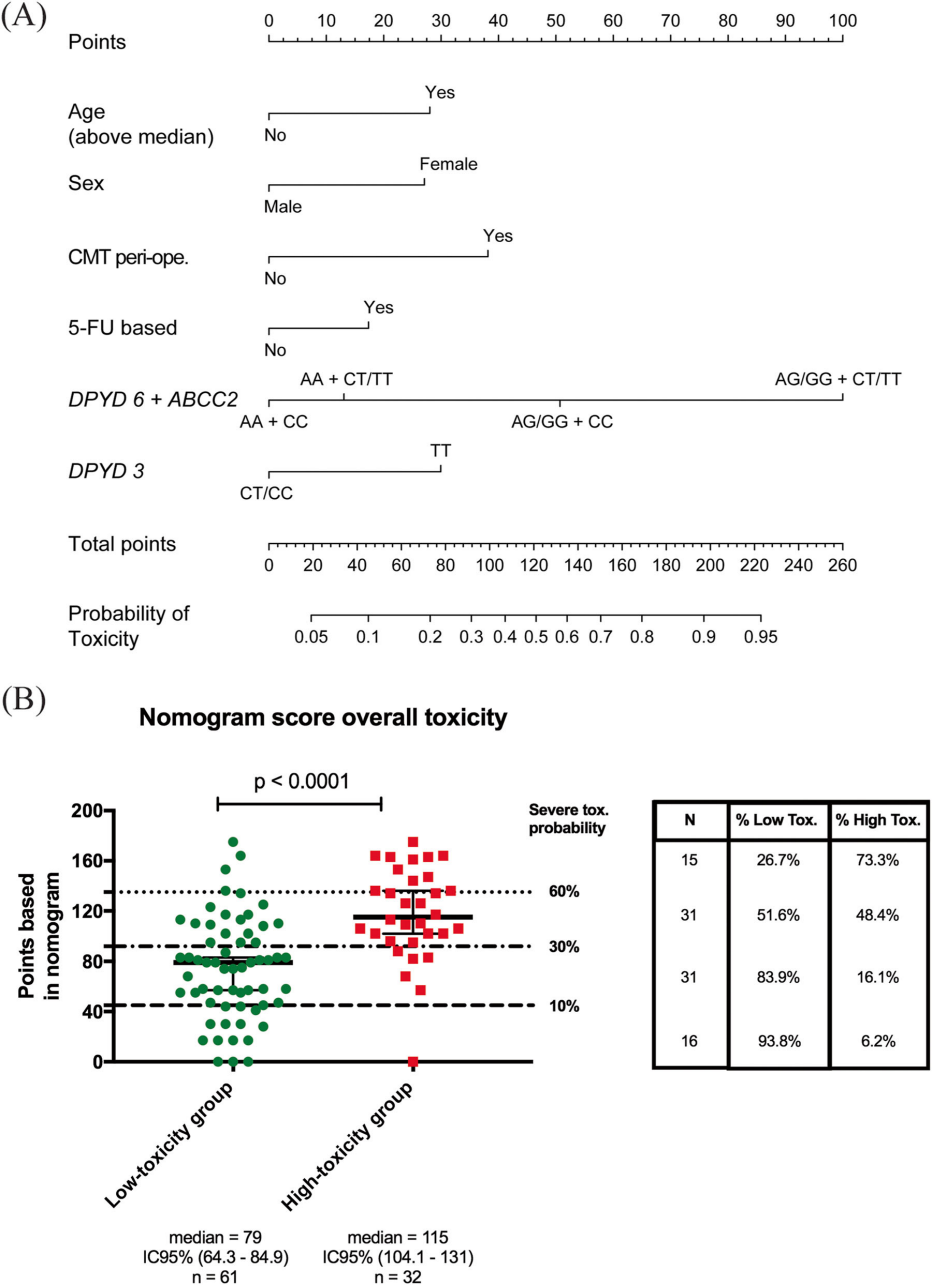
Nomogram for estimating overall toxicity risk based on the multifactorial model 4. **A** The nomogram was developed on the basis of the final multivariate logistic regression model. **B** The total sum of points of low and high-toxicity groups is shown in the scatter plot on the left. Broken lines represent the probability of developing severe toxicity according to the points. On the right side, the percentage of low and high-toxicity groups is shown according to the probability estimated in the nomogram*. Low Tox. Low-toxicity group; High Tox. High-toxicity group. DPYD 6, rs1801265; DPYD 3, rs1801159 (in nomogram). Significance: P < 0.05*

As an approximation for future validation of our results we developed a multivariate logistic regression-based nomogram that estimates the probability of a given patient to experience grade 3–4 overall general toxicity (Fig. 2A). This model is well calibrated (Supplementary Fig. S2) and has an acceptable discriminatory capacity, with an optimism-corrected c-index of 0.72 (95% CI, 0.72–0.92). Figure 2B shows the distribution of nomogram values for each patient. As expected, the median values for low or high-toxicity groups were significantly different (*p* < 0.0001). In addition, we established a different cut-off according to the points on delivered by the nomogram. Thereby ≤45-point patients have a 10% probability of developing toxicity. Encouragingly, 93% of patients in the lower range group correspond to the low-toxicity group. In contrast, patients with > 136-accumulated points are at a higher risk to develop toxicity and 73% of them are in the high-toxicity group.

## Discussion

Chemotherapy treatment-related toxicity remains a critical problem in GC patients. Unfortunately, the relative benefit in terms of patient survival associated with targeted therapies is rather modest [52–55]. Therefore, optimizing chemotherapy regimens becomes crucial to improve GC patient survival. Within this context, the elaboration of reliable models that predict treatment related toxicities might ensure patient safety in interventional studies. While guidelines exist for genotypes relating to fluoropyrimidine toxicity, our study also demonstrates associations related to platinum presence. The Clinical Pharmacogenetics Implementation Consortium has delivered guidelines for the clinical interpretation of four *DPYD* genotypes related to severe fluoropyrimidine toxicity within European populations [56]. However, while the frequency of these single nucleotide polymorphisms (SNPs) could reach ~ 10% in some European populations the reported frequency in the Latin-American populations is below 0.7% [57] (Suarez-Kurtz 2020; Nugent et al. 2019). This may be primarily due to the underrepresentation of the Latin-American population in genetic studies [58]. In accordance, the SNPs in DPYD (rs55886062), included in the CPIC guide, were not mutated homozygous nor were the heterozygous genotypes detected in the Chilean patients used in this study (data not shown). Given the world population that is not derived from European ancestry, the identification of new associations between the genome and fluoropyrimidine and platinum toxicity is of the utmost importance and may complement the current CPIC guidelines once further validation has been completed. Our findings are one of first to frame toxicity pharmacogenetics in an underexplored Latin American population and given the inherent global differences in SNP distribution, it is not beyond the realms of imagination to envisage that future pharmacogenetic tests are applied in a regional or populational manner.

In line with previous GC reports, patients in our cohort were predominantly males [59, 60] and advanced stage [42, 60, 61]. Similarly, median overall survival, histological type and overall toxicity were in agreement with the current literature (Supplementary Table S1 and Supplementary Fig. S1). Regarding age at diagnosis, the association with combined chemotherapy toxicity is probably explained by the age of recruited participants in most GC-trials that range between 50 and 60 years [62]. Interestingly, the inclusion of age in our final model increased the predictive power. A study reported no significant differences in the incidence of grade 3–4 toxicities in gastro-esophageal cancer patients comparing ≥70 vs < 70 year-old participants [63]. However, in many cases a higher prevalence of toxicity in older patients leads to chemotherapy discontinuation [64]. In contrast, a pooled analysis concluded that chemotherapy-related serious adverse events were significantly higher in > 65 year-old patients [65]. Accordingly, a recent study demonstrated that older GC patients (≥ 70) experience more severe toxicities versus younger patients [66]. A number of studies, including meta-analyses, have shown an increased risk of severe toxicity associated to fluoropyrimidine/platinum-based chemotherapy in female gastric and colorectal cancer patients [7, 36, 37, 67]. In accordance, we found a trend towards higher toxicity among females in our study (Table 2).

Compared to intravenous 5-FU, oral capecitabine (5-FU pro-drug) increases OS and response rates in combination with platinum compounds. Also, 5-FU/cisplatin is associated with greater toxicity [68–70]. In line with these findings, we observed that incorporation of certain regimens improved the predictive power of our models (Table 3). A recent study in colorectal cancer patients demonstrated that FOLFOX was associated with a significant increase in stomatitis and neutropenia, but decreased diarrhea and hand-foot syndrome versus CAPEOX [71].

Our model 2 includes the most relevant associations between selected SNPs and overall toxicity (Table 4). Several reports confirm DPYD is a reaction-limiting enzyme for 5-FU catabolism. In fact, DPYD-deficiency is commonly associated with a lower drug-clearance and increased toxicity [72]. In our analysis, AG/GG *DPYD* (rs1801265) genotypes were associated with higher grade 3–4 toxicity (OR = 4.20, *p* = 0.002). This variant causes a Cys 29 to Arg substitution that reduces DPYD enzymatic activity and increases 5-FU-related toxicity [73]. Functional studies demonstrate that AG and GG genotypes of *DPYD* (rs1801265) have a significantly lower 5-FU degradation rates (5-FUDR) compared to AA, with a profound effect for GG [73]. Likewise, the CT *DPYD* (rs2297595) genotype was associated with grade 3–4 toxicity versus TT, although individually was not significant (OR = 2.71, *p* = 0.21). The same study reported that the CT *DPYD* (rs2297595) genotype had a significantly lower 5-FUDR versus TT [73]. On the other hand, the TC/CC *DPYD* (rs1801159) genotypes were associated with a lower probability of grade 3–4 overall toxicity versus the TT genotype (OR = 0.45, *p* = 0.071). However, studies on this polymorphism are somewhat inconsistent and some have reported an association with increased severe toxicity [28, 74, 75] or no association [76–78]. A potential explanation for our finding is the high frequency of the C allele in this subset, reaching 34% (Supplementary Table S8). In sharp contrast, European cohorts report 18.3% for the C allele (*n* = 157) [73]. Similarly, an Asian study reports a 27% frequency (*n* = 362) [75]. Notably, C allele frequency in the American population is 27%, whereas in East Asian, European, African and South Asian populations is 27, 19, 15 and 8% respectively [32]. Therefore, differences can be attributed to specific geographical/ethnic factors.

Our study also found an association between *GSTP1*/*ERCC2* SNPs and overall toxicity. These are linked to the formation of DNA-adducts. The AG/GG *GSTP1* (rs1695) genotypes were associated with a lower probability of grade 3–4 toxicity compared to AA. This “protective” role of the G allele has been previously reported in gastro-esophageal [79], colorectal [78], ovarian [80], testicular [41] and lung cancer [81]. A potential mechanism to explain this protective role could be the activation of the JNK pathway [79, 82] that increases cell defense mechanisms. Conversely, GT/GG genotypes in *ERCC2* (rs13181) were associated with a higher probability of grade 3–4 overall toxicity (Table 4). These polymorphic variants decrease repair efficacy and may thus increase DNA adducts [83, 84]. This suggests that increased toxicity may be mediated by platinum damage to normal cells [85].

In line with previous publications, our study found a significant association between *DPYD* (rs1801265) SNPs and grade 3–4 overall toxicity only in male patients (Supplementary Table S4), [7]. Our paired-SNP analysis found a strong association between *DPYD* (rs1801265) and *ABCC2* (rs717620) SNPs and overall toxicity (see Table 5). In particular, AG/GG (*DPYD*) and CT/TT (*ABCC2*) patients had a high probability of developing grade 3–4 overall toxicity (OR = 11.25, 95% CI = 1.25–245.45). The ABCC2 (rs717620) polymorphism is located in the promoter region of the gene, and has been previously associated with decreased protein expression in vitro [86]; *ABCC2* also mediates the export/elimination of glutathione-oxaliplatin conjugates [87] therefore an impaired function could decrease export of the drug leading to toxicity. Previous studies in colorectal and lung cancer [88, 89] have associated this polymorphism to severe fluoropyrimidine/platinum-related hematological toxicity. Thus, the *DPYD* (rs1801265)/*ABCC2* (rs717620) SNP combination could potentiate fluoropyrimidines and/or oxaliplatin derived toxicities. Again, given the SNP frequency in our analysis, this finding requires further validation by a larger cohort.

Utilizing a classification algorithm that involved 28 SNPs and 1 clinical variable (histology) a study by Yin et al. reported that the best prediction of toxicity was achieved in lung cancer patients that received platinum-based therapies [39]. Moreover, these authors demonstrated that the *ABCG2* rs2231142-*CES5A* rs3859104 SNPs combination was strongly associated with grade 3–4 platinum toxicity (adjusted OR = 8.044, *p* = 4.350 × 10–5) [90]. In this regard, our models displayed better adjustments (based on Pseudo R^2^) after adding clinical/treatment factors and SNPs (models 3 and 4, Table 6). Our Model 4 was the best-fitted model in terms of sensitivity, specificity, accuracy and AUC (Fig. 1D). Previous studies have used also this strategy with consistent results [7, 40, 41, 91].

The frequency of grade 3–4 toxicity is observed in only 10–15% of gastric cancer patients as medical oncologists often make alterations to treatment protocols when lower toxicities start to manifest. The number of cases and controls incorporated into this study allowed statistically significant differences to be observed, however despite over 223 medical records being screened, we recognize as a limitation that the number of patients was a limiting factor in further interpreting our data and thus validation in a larger cohort is required before these models can be considered in a clinical setting. A future cohort will permit better precision analysis of the combinatorial SNPs. Certain SNPs, despite not achieving the individual significance standard of *p* < 0.05, were included in the models due to their influence or effect on the development of severe toxicity has been previously reported, or their inclusion improved the Pseudo R^2^ values. Interestingly, according to the CPIC guide, the polymorphisms for DPYD rs1801265 (also known as DPYD*9A) and DPYD rs1801159 (also known as DPYD*5), which were both incorporated into our model, are classified to not affect the DPD function in a clinically relevant manner in the context of 5-fluorouracil related toxicity [30]. Interestingly, in accordance with our results, recent reports have shown an association with severe toxicity and high levels of 5-FU post-treatment for DPYD-rs1801265 [92–94].

A further option is a model / nomogram comparison with other similar studies. Schwab et al. [7] and Botticelli et al. [95] previously proposed nomograms to predict toxicity to 5-FU, and thus could be incorporated as a reference in future validation studies. A potential comment on this study could be the heterogeneity of treatments received by patients and that a prospective study considering a limited number of regimens would allow analyses the actual doses and duration of chemotherapy as potential variables associated with toxicity. While this is true, our gastric cancer patients and their treatments are a reflection of standard clinical practice. This heterogeneity in treatment was present despite the patients being part of the same recruiting clinical center and being treated by the same group of medical oncologists. Thus, this heterogeneity observed among our fluoropyrimidine and platinum-based treatments will always exist in the oncology clinic and thus any predictive model or algorithm will be required to be effective in face of this variable. The real-world treatment and clinical outcomes in this study have allowed a “proof of concept” of a model which integrates clinical and pharmacogenetic (three SNPs in two different genes) variables to improve the prediction of toxicity associated with fluoropyrimidine and platinum-based chemotherapy. Furthermore, recent studies have demonstrated that genetic elements outside the coding region of genes are potential regulators of pharmacokinetic and pharmacodynamic processes. In the *TYMS* gene, variants in UTR regions of 5’VNTR 28 bp-repeat (rs45445694) and 3’UTR 6 bp-indel (rs11280056) have been associated with severe toxicity in patients receiving fluoropyrimidine based treatments [77]. Furthermore, regulatory molecules of the non-coding RNA type, such as circular RNA (circRNAs) and Long Non-Coding RNA (lncRNAs), have been correlated to clinical variables (TNM stage, presence of metastasis and diagnosis) in patients with GC [96]. Interestingly, polymorphisms in lncRNAs of ANRIL (rs1333049) and MEG3 (rs116907618) genes were associated with severe overall and gastrointestinal toxicity in patients with lung cancer treated with platinum-based chemotherapy [97]. In addition, another regulator of gene expression are the Micro RNA (miRNAs), where genetic variations in miRNA binding sites are associated with an altered drug response [98]. In a similar vein, a recent publication by Powell et al. reported the mapping of miRNA-mRNA interactions in several pharmacogenes, in fact, the authors identified an hsa-mir-27b-DPYD interaction at a previously validated binding site, which may suggest the existence of additional elements that contribute to the individualism of drug response [99].

Given that the purpose of study is to predict toxicity to chemotherapy, it would be interesting in further validation studies to test our proposed clinical variables and individual and combinations of SNPs, together with emerging variables such as changes in expression, sequence, and binding sites of non-coding RNAs. This may give us a more complete picture on how to predict severe toxicity associated with chemotherapy and thus improve patient quality of life and survival.

## Conclusions

In summary, in the absence of reliable markers and clinically relevant models to predict patient toxicity derived from fluoropyrimidine/platinum-based chemotherapy, herein we present for future validation a logistic regression-based model that integrates clinical, treatment and common SNPs.

## Supplementary Information

**Additional file 1: Supplementary Material S1.Supplementary Fig. S1.** Overall survival rates in the study cohort, **Supplementary Fig. S2.** Calibration plot for the prognostic model, **Supplementary Table S1.** Demographic and clinic-pathological characteristics of study population (*N* = 93), **Supplementary Table S2.** Platinum plus fluoropyrimidine-based chemotherapy combined treatments used in gastric cancer patients (N = 93), **Supplementary Table S3.** Grades of toxicity in gastric cancer patients by the Common Toxicity Criteria for Adverse Events (CTCAE) 4.0, **Supplementary Table S4.** Sex subgroup association analysis of the SNPs *DPYD* (rs1801265). **Supplementary Table S5.** Models for hematological grade ≥ 3 toxicity in gastric cancer patients treated with platinum/fluoropyridines -based chemotherapy using multivariate analysis. **Supplementary Table S6.** Models for gastrointestinal grade ≥ 3 toxicity in gastric cancer patients treated with platinum/fluoropyridines -based chemotherapy using multivariate analysis. **Supplementary Table S7.** Models for neurological grade ≥ 3 toxicity in gastric cancer patients treated with platinum/fluoropyridines -based chemotherapy using multivariate analysis. **Supplementary Table S8.** Genotypic and allelic frequencies for the analyzed polymorphisms, **Supplementary Table S9.** ID assay for each of the analyzed polymorphisms. **Supplementary Table S10.** SNPs selection based in score for fluoropyrimidines. **Supplementary Table S11.** Final score for fluoropyrimidines. **Supplementary Table S12.** SNPs selection based in score for platinums. **Supplementary Table S13.** Final score for platinums. **Supplementary Table S14.** Sensitivity, specificity and accuracy calculations from a 2 × 2 confusion matrix. **Supplementary Methods:** Details of chemotherapy schemes, SNPs selection and classifications algorithm used. **Supplementary Data File 1.** All raw data used in this study.

## Data Availability

All data generated or analyzed during this study are included in this published article and it supplementary information files.

## References

[1] Bray F, Ferlay J, Soerjomataram I, Siegel RL, Torre LA, Jemal A (2018). Global cancer statistics 2018: GLOBOCAN estimates of incidence and mortality worldwide for 36 cancers in 185 countries. CA Cancer J Clin.

[2] Ferro A, Peleteiro B, Malvezzi M, Bosetti C, Bertuccio P, Levi F, Negri E, la Vecchia C, Lunet N (2014). Worldwide trends in gastric cancer mortality (1980-2011), with predictions to 2015, and incidence by subtype. Eur J Cancer.

[3] de la Jara JJ, Bastias G, Ferreccio C, Moscoso C, Sagues S, Cid C (2015). A snapshot of cancer in Chile: analytical frameworks for developing a cancer policy. Biol Res..

[4] Owen GI, Pinto MP, Retamal IN, Fernádez MF, Cisternas B, Mondaca S (2018). Chilean Gastric Cancer Task Force: A study protocol to obtain a clinical and molecular classification of a cohort of gastric cancer patients. Medicine (Baltimore).

[5] Carmona-Bayonas A, Jiménez-Fonseca P, Lorenzo MLS, Ramchandani A, Martínez EA, Custodio A, Garrido M, Echavarría I, Cano JM, Barreto JEL, García TG, Manceñido FÁ, Lacalle A, Cardona MF, Mangas M, Visa L, Buxó E, Azkarate A, Díaz-Serrano A, Montes AF, Rivera F (2016). On the effect of triplet or doublet chemotherapy in advanced gastric Cancer: results from a National Cancer Registry. J Natl Compr Cancer Netw.

[6] Park H, Jin RU, Wang-Gillam A, Suresh R, Rigden C, Amin M, Tan BR, Pedersen KS, Lim KH, Trikalinos NA, Acharya A, Copsey ML, Navo KA, Morton AE, Gao F, Lockhart AC (2020). FOLFIRINOX for the treatment of advanced Gastroesophageal cancers: a phase 2 nonrandomized clinical trial. JAMA Oncol.

[7] Schwab M, Zanger UM, Marx C, Schaeffeler E, Klein K, Dippon J, Kerb R, Blievernicht J, Fischer J, Hofmann U, Bokemeyer C, Eichelbaum M, German 5-FU Toxicity Study Group (2008). Role of genetic and nongenetic factors for fluorouracil treatment-related severe toxicity: a prospective clinical trial by the German 5-FU toxicity study group. J Clin Oncol.

[8] Lopez Sobella M, Criado Illana MT, Esteban Herrera B, Lopez Arranza MC (2008). Severe 5-fluorouracil induced toxicity associated with dihydropyrimidine dehydrogenase deficiency. Farm Hosp.

[9] Pachman DR, Qin R, Seisler DK, Smith EM, Beutler AS, Ta LE (2015). Clinical course of Oxaliplatin-induced neuropathy: results from the randomized phase III trial N08CB (alliance). J Clin Oncol.

[10] Erichsen HC, Chanock SJ (2004). SNPs in cancer research and treatment. Br J Cancer.

[11] Evans WE, Relling MV (1999). Pharmacogenomics: translating functional genomics into rational therapeutics. Science.

[12] López-Cortés A, Guerrero S, Redal MA, Alvarado AT, Quiñones LA. State of art of Cancer pharmacogenomics in Latin American populations. Int J Mol Sci. 2017;18(6). 10.3390/ijms18060639. 10.3390/ijms18060639 PMC5485925 28545225

[13] Quiñones L, Roco Á, Cayún JP, Escalante P, Miranda C, Varela N, Meneses F, Gallegos B, Zaruma-Torres F, Lares-Asseff I (2017). Clinical applications of pharmacogenomics. Rev Med Chil.

[14] Relling MV, Evans WE (2015). Pharmacogenomics in the clinic. Nature..

[15] Wang D, Lippard SJ (2005). Cellular processing of platinum anticancer drugs. Nat Rev Drug Discov.

[16] Sakaeda T, Nakamura T, Okumura K (2002). MDR1 genotype-related pharmacokinetics and pharmacodynamics. Biol Pharm Bull.

[17] Scripture CD, Figg WD (2006). Drug interactions in cancer therapy. Nat Rev Cancer.

[18] Sun Y, Pan J, Tong X, Chen E, Yan W, Wu M, Qu Q, Qu J (2019). Glutathione S-transferases genes variants and chemotherapy efficacy in gastrointestinal cancer patients: a meta-analysis based on 50 pharmacogenetic studies. J Cancer.

[19] Marsh S, McLeod H, Dolan E, Shukla SJ, Rabik CA, Gong L, Hernandez-Boussard T, Lou XJ, Klein TE, Altman RB (2009). Platinum pathway. Pharmacogenet Genomics.

[20] Palmirotta R, Carella C, Silvestris E, Cives M, Stucci SL, Tucci M (2018). SNPs in predicting clinical efficacy and toxicity of chemotherapy: walking through the quicksand. Oncotarget.

[21] Roco A, Cayún J, Contreras S, Stojanova J, Quiñones L (2014). Can pharmacogenetics explain efficacy and safety of cisplatin pharmacotherapy?. Front Genet.

[22] Arnould S, Hennebelle I, Canal P, Bugat R, Guichard S (2003). Cellular determinants of oxaliplatin sensitivity in colon cancer cell lines. Eur J Cancer.

[23] Longley DB, Harkin DP, Johnston PG (2003). 5-fluorouracil: mechanisms of action and clinical strategies. Nat Rev Cancer.

[24] Panczyk M (2014). Pharmacogenetics research on chemotherapy resistance in colorectal cancer over the last 20 years. World J Gastroenterol.

[25] Diasio RB, Harris BE (1989). Clinical pharmacology of 5-fluorouracil. Clin Pharmacokinet.

[26] Dhelens C, Bonadona A, Thomas F, Chapuis C, Potton L, Marsili S, Bedouch P, Schwebel C (2016). Lethal 5-fluorouracil toxicity in a colorectal patient with severe dihydropyrimidine dehydrogenase (DPD) deficiency. Int J Color Dis.

[27] Van Kuilenburg AB, Meinsma R, Zoetekouw L, Van Gennip AH (2002). Increased risk of grade IV neutropenia after administration of 5-fluorouracil due to a dihydropyrimidine dehydrogenase deficiency: high prevalence of the IVS14+1g>a mutation. Int J Cancer.

[28] Maring JG, van Kuilenburg AB, Haasjes J, Piersma H, Groen HJ, Uges DR (2002). Reduced 5-FU clearance in a patient with low DPD activity due to heterozygosity for a mutant allele of the DPYD gene. Br J Cancer.

[29] Jakobsen A, Nielsen JN, Gyldenkerne N, Lindeberg J (2005). Thymidylate synthase and methylenetetrahydrofolate reductase gene polymorphism in normal tissue as predictors of fluorouracil sensitivity. J Clin Oncol.

[30] Amstutz U, Henricks LM, Offer SM, Barbarino J, Schellens JHM, Swen JJ, Klein TE, McLeod HL, Caudle KE, Diasio RB, Schwab M (2018). Clinical Pharmacogenetics implementation consortium (CPIC) guideline for Dihydropyrimidine dehydrogenase genotype and Fluoropyrimidine dosing: 2017 update. Clin Pharmacol Ther.

[31] Karczewski KJ, Francioli LC, Tiao G, Cummings BB, Alföldi J, Wang Q, Collins RL, Laricchia KM, Ganna A, Birnbaum DP, Gauthier LD, Brand H, Solomonson M, Watts NA, Rhodes D, Singer-Berk M, England EM, Seaby EG, Kosmicki JA, Walters RK, Tashman K, Farjoun Y, Banks E, Poterba T, Wang A, Seed C, Whiffin N, Chong JX, Samocha KE, Pierce-Hoffman E, Zappala Z, O’Donnell-Luria AH, Minikel EV, Weisburd B, Lek M, Ware JS, Vittal C, Armean IM, Bergelson L, Cibulskis K, Connolly KM, Covarrubias M, Donnelly S, Ferriera S, Gabriel S, Gentry J, Gupta N, Jeandet T, Kaplan D, Llanwarne C, Munshi R, Novod S, Petrillo N, Roazen D, Ruano-Rubio V, Saltzman A, Schleicher M, Soto J, Tibbetts K, Tolonen C, Wade G, Talkowski ME, Aguilar Salinas CA, Ahmad T, Albert CM, Ardissino D, Atzmon G, Barnard J, Beaugerie L, Benjamin EJ, Boehnke M, Bonnycastle LL, Bottinger EP, Bowden DW, Bown MJ, Chambers JC, Chan JC, Chasman D, Cho J, Chung MK, Cohen B, Correa A, Dabelea D, Daly MJ, Darbar D, Duggirala R, Dupuis J, Ellinor PT, Elosua R, Erdmann J, Esko T, Färkkilä M, Florez J, Franke A, Getz G, Glaser B, Glatt SJ, Goldstein D, Gonzalez C, Groop L, Haiman C, Hanis C, Harms M, Hiltunen M, Holi MM, Hultman CM, Kallela M, Kaprio J, Kathiresan S, Kim BJ, Kim YJ, Kirov G, Kooner J, Koskinen S, Krumholz HM, Kugathasan S, Kwak SH, Laakso M, Lehtimäki T, Loos RJF, Lubitz SA, Ma RCW, MacArthur DG, Marrugat J, Mattila KM, McCarroll S, McCarthy MI, McGovern D, McPherson R, Meigs JB, Melander O, Metspalu A, Neale BM, Nilsson PM, O’Donovan MC, Ongur D, Orozco L, Owen MJ, Palmer CNA, Palotie A, Park KS, Pato C, Pulver AE, Rahman N, Remes AM, Rioux JD, Ripatti S, Roden DM, Saleheen D, Salomaa V, Samani NJ, Scharf J, Schunkert H, Shoemaker MB, Sklar P, Soininen H, Sokol H, Spector T, Sullivan PF, Suvisaari J, Tai ES, Teo YY, Tiinamaija T, Tsuang M, Turner D, Tusie-Luna T, Vartiainen E, Vawter MP, Ware JS, Watkins H, Weersma RK, Wessman M, Wilson JG, Xavier RJ, Neale BM, Daly MJ, MacArthur DG, Genome Aggregation Database Consortium (2020). The mutational constraint spectrum quantified from variation in 141,456 humans. Nature..

[32] Auton A, Brooks LD, Durbin RM, Garrison EP, Kang HM, Korbel JO (2015). A global reference for human genetic variation. Nature..

[33] Shimoyama S (2009). Pharmacogenetics of fluoropyrimidine and cisplatin. A future application to gastric cancer treatment. J Gastroenterol Hepatol.

[34] Toffoli G, Cecchin E (2007). Pharmacogenetics and stomach cancer: an update. Pharmacogenomics..

[35] Patel JN, Fuchs CS, Owzar K, Chen Z, McLeod HL (2013). Gastric cancer pharmacogenetics: progress or old tripe?. Pharmacogenomics..

[36] Milano G, Etienne MC, Cassuto-Viguier E, Thyss A, Santini J, Frenay M, Renee N, Schneider M, Demard F (1992). Influence of sex and age on fluorouracil clearance. J Clin Oncol Off J Am Soc Clin Oncol.

[37] Sloan JA, Goldberg RM, Sargent DJ, Vargas-Chanes D, Nair S, Cha SS, Novotny PJ, Poon MA, O’Connell MJ, Loprinzi CL (2002). Women experience greater toxicity with fluorouracil-based chemotherapy for colorectal cancer. J Clin Oncol Off J Am Soc Clin Oncol.

[38] Davidson M, Wagner AD, Kouvelakis K, Nanji H, Starling N, Chau I, Watkins D, Rao S, Peckitt C, Cunningham D (2019). Influence of sex on chemotherapy efficacy and toxicity in oesophagogastric cancer: a pooled analysis of four randomised trials. Eur J Cancer.

[39] Yin J-Y, Li X, Li X-P, Xiao L, Zheng W, Chen J, Mao CX, Fang C, Cui JJ, Guo CX, Zhang W, Gao Y, Zhang CF, Chen ZH, Zhou H, Zhou HH, Liu ZQ (2016). Prediction models for platinum-based chemotherapy response and toxicity in advanced NSCLC patients. Cancer Lett.

[40] Anandi P, Dickson AL, Feng Q, Wei W-Q, Dupont WD, Plummer D, et al. Combining clinical and candidate gene data into a risk score for azathioprine-associated leukopenia in routine clinical practice. Pharmacogenomics J. 2020. 10.1038/s41397-020-0163-4. 10.1038/s41397-020-0163-4 PMC7426242 32054992

[41] Lavanderos MA, Cayún JP, Roco Á, Sandoval C, Cerpa L, Rubilar JC, Cerro R, Molina-Mellico S, Celedón C, Cerda B, García-Martín E, Agúndez JAG, Acevedo C, Peña K, Cáceres DD, Varela NM, Quiñones LA (2019). Association study among candidate genetic polymorphisms and chemotherapy-related severe toxicity in testicular Cancer patients. Frontiers in Pharmacology.

[42] Cordova-Delgado M, Pinto MP, Retamal IN, Munoz-Medel M, Bravo ML, Fernandez MF, et al. High Proportion of Potential Candidates for Immunotherapy in a Chilean Cohort of Gastric Cancer Patients: Results of the FORCE1 Study. Cancers (Basel). 2019;11. 10.3390/cancers11091275. 10.3390/cancers11091275 PMC6770659 31480291

[43] Sole X, Guino E, Valls J, Iniesta R, Moreno V (2006). SNPStats: a web tool for the analysis of association studies. Bioinformatics..

[44] Menard S (2000). Coefficients of determination for multiple logistic regression analysis. Am Stat.

[45] Han J, Kamber M, Pei J, JBT-DM P, Third E, Han J, Kamber M (2012). 8 - Classification: Basic Concepts. The Morgan Kaufmann Series in Data Management Systems.

[46] Kuhn M. Building Predictive Models in R Using the caret Package. J Stat Software. 2008;1(5). 10.18637/jss.v028.i05.

[47] Zhang Z, Kattan MW (2017). Drawing Nomograms with R: applications to categorical outcome and survival data. Ann Transl Med.

[48] Whirl-Carrillo M, McDonagh EM, Hebert JM, Gong L, Sangkuhl K, Thorn CF (2012). Pharmacogenomics knowledge for personalized medicine. Clin Pharmacol Ther.

[49] Sunyaev S, Ramensky V, Koch I, Lathe W, Kondrashov AS, Bork P (2001). Prediction of deleterious human alleles. Hum Mol Genet.

[50] Ng PC, Henikoff S (2003). SIFT: predicting amino acid changes that affect protein function. Nucleic Acids Res.

[51] Akaike H (1974). A new look at the statistical model identification. IEEE Trans Automat Contr.

[52] Bang Y-J, Van Cutsem E, Feyereislova A, Chung HC, Shen L, Sawaki A (2010). Trastuzumab in combination with chemotherapy versus chemotherapy alone for treatment of HER2-positive advanced gastric or gastro-oesophageal junction cancer (ToGA): a phase 3, open-label, randomised controlled trial. Lancet.

[53] Fuchs CS, Tomasek J, Yong CJ, Dumitru F, Passalacqua R, Goswami C (2014). Ramucirumab monotherapy for previously treated advanced gastric or gastro-oesophageal junction adenocarcinoma (REGARD): an international, randomised, multicentre, placebo-controlled, phase 3 trial. Lancet.

[54] Wilke H, Muro K, Van Cutsem E, Oh S-C, Bodoky G, Shimada Y (2014). Ramucirumab plus paclitaxel versus placebo plus paclitaxel in patients with previously treated advanced gastric or gastro-oesophageal junction adenocarcinoma (RAINBOW): a double-blind, randomised phase 3 trial. Lancet Oncol.

[55] Kang Y-K, Boku N, Satoh T, Ryu M-H, Chao Y, Kato K (2017). Nivolumab in patients with advanced gastric or gastro-oesophageal junction cancer refractory to, or intolerant of, at least two previous chemotherapy regimens (ONO-4538-12, ATTRACTION-2): a randomised, double-blind, placebo-controlled, phase 3 trial. Lancet.

[56] Wörmann B, Bokemeyer C, Burmeister T, Köhne C-H, Schwab M, Arnold D, Blohmer JU, Borner M, Brucker S, Cascorbi I, Decker T, de Wit M, Dietz A, Einsele H, Eisterer W, Folprecht G, Hilbe W, Hoffmann J, Knauf W, Kunzmann V, Largiadèr CR, Lorenzen S, Lüftner D, Moehler M, Nöthen MM, Pox C, Reinacher-Schick A, Scharl A, Schlegelberger B, Seufferlein T, Sinn M, Stroth M, Tamm I, Trümper L, Wilhelm M, Wöll E, Hofheinz RD (2020). Dihydropyrimidine dehydrogenase testing prior to treatment with 5-fluorouracil, Capecitabine, and Tegafur: a consensus paper. Oncol Res Treat.

[57] Zhou Y, Dagli Hernandez C, Lauschke VM (2020). Population-scale predictions of DPD and TPMT phenotypes using a quantitative pharmacogene-specific ensemble classifier. Br J Cancer.

[58] Nugent A, Conatser KR, Turner LL, Nugent JT, Sarino EMB, Ricks-Santi LJ (2019). Reporting of race in genome and exome sequencing studies of cancer: a scoping review of the literature. Genet Med.

[59] Okada E, Ukawa S, Nakamura K, Hirata M, Nagai A, Matsuda K, Ninomiya T, Kiyohara Y, Muto K, Kamatani Y, Yamagata Z, Kubo M, Nakamura Y, Tamakoshi A, Shimoyama R, Makimoto S, Harada H, Fujikawa T, Minami S, Uchida E, Miyashita M, Kajiyama Y, Tomita N, Nagahara A, Asai S, Moriyama M, Takahashi Y, Fujioka T, Obara W, Mori S, Ito H, Nagayama S, Miki Y, Masumoto A, Yamada A, Nishizawa Y, Kodama K, Ban H, Murata S, Koretsune Y, Hirao M, Ogata H (2017). Demographic and lifestyle factors and survival among patients with esophageal and gastric cancer: the biobank Japan project. J Epidemiol.

[60] Gonzalez-Hormazabal P, Musleh M, Bustamante M, Stambuk J, Pisano R, Valladares H, et al. Polymorphisms in RAS/RAF/MEK/ERK Pathway Are Associated with Gastric Cancer. Genes (Basel). 2018;10. 10.3390/genes10010020. 10.3390/genes10010020 PMC6356706 30597917

[61] Zong L, Abe M, Seto Y, Ji J (2016). The challenge of screening for early gastric cancer in China. Lancet.

[62] Wagner AD, Grothe W, Haerting J, Kleber G, Grothey A, Fleig WE (2006). Chemotherapy in advanced gastric cancer: a systematic review and meta-analysis based on aggregate data. J Clin Oncol Off J Am Soc Clin Oncol.

[63] Trumper M, Ross PJ, Cunningham D, Norman AR, Hawkins R, Seymour M, Harper P, Iveson T, Nicolson M, Hickish T (2006). Efficacy and tolerability of chemotherapy in elderly patients with advanced oesophago-gastric cancer: a pooled analysis of three clinical trials. Eur J Cancer.

[64] Visa L, Jiménez-Fonseca P, Martínez EA, Hernández R, Custodio A, Garrido M, Viudez A, Buxo E, Echavarria I, Cano JM, Macias I, Mangas M, de Castro EM, García T, Manceñido FÁ, Montes AF, Azkarate A, Longo F, Serrano AD, López C, Hurtado A, Cerdá P, Serrano R, Gil-Negrete A, Carnicero AM, Pimentel P, Ramchandani A, Carmona-Bayonas A, AGAMENON Study Group (2018). Efficacy and safety of chemotherapy in older versus non-older patients with advanced gastric cancer: a real-world data, non-inferiority analysis. J Geriatr Oncol.

[65] Jatoi A, Foster NR, Egner JR, Burch PA, Stella PJ, Rubin J, Dakhil SR, Sargent DJ, Murphy BR, Alberts SR (2010). Older versus younger patients with metastatic adenocarcinoma of the esophagus, gastroesophageal junction, and stomach: a pooled analysis of eight consecutive north central Cancer treatment group (NCCTG) trials. Int J Oncol.

[66] Slagter AE, Tudela B, van Amelsfoort RM, Sikorska K, van Sandick JW, van de Velde CJH, van Grieken NCT, Lind P, Nordsmark M, Putter H, Hulshof MCCM, van Laarhoven HWM, Grootscholten C, Braak JPBM, Meershoek-Klein Kranenbarg E, Jansen EPM, Cats A, Verheij M (2020). Older versus younger adults with gastric cancer receiving perioperative treatment: results from the CRITICS trial. Eur J Cancer.

[67] Cristina V, Mahachie J, Mauer M, Buclin T, Van Cutsem E, Roth A (2018). Association of Patient sex with Chemotherapy-Related Toxic Effects: a retrospective analysis of the PETACC-3 trial conducted by the EORTC gastrointestinal group. JAMA Oncol..

[68] Cunningham D, Starling N, Rao S, Iveson T, Nicolson M, Coxon F, Middleton G, Daniel F, Oates J, Norman AR (2008). Capecitabine and oxaliplatin for advanced esophagogastric cancer. N Engl J Med.

[69] Kang Y-K, Kang W-K, Shin D-B, Chen J, Xiong J, Wang J, Lichinitser M, Guan Z, Khasanov R, Zheng L, Philco-Salas M, Suarez T, Santamaria J, Forster G, McCloud PI (2009). Capecitabine/cisplatin versus 5-fluorouracil/cisplatin as first-line therapy in patients with advanced gastric cancer: a randomised phase III noninferiority trial. Ann Oncol Off J Eur Soc Med Oncol.

[70] Lordick F, Lorenzen S, Yamada Y, Ilson D (2014). Optimal chemotherapy for advanced gastric cancer: is there a global consensus?. Gastric Cancer.

[71] Loree JM, Sha A, Soleimani M, Kennecke HF, Ho MY, Cheung WY, Mulder KE, Abadi S, Spratlin JL, Gill S (2018). Survival impact of CAPOX versus FOLFOX in the adjuvant treatment of stage III Colon Cancer. Clin Colorectal Cancer.

[72] Del Re M, Di Paolo A, van Schaik RH, Bocci G, Simi P, Falcone A (2010). Dihydropyrimidine dehydrogenase polymorphisms and fluoropyrimidine toxicity: ready for routine clinical application within personalized medicine?. EPMA J.

[73] Gentile G, Botticelli A, Lionetto L, Mazzuca F, Simmaco M, Marchetti P, Borro M (2016). Genotype–phenotype correlations in 5-fluorouracil metabolism: a candidate DPYD haplotype to improve toxicity prediction. Pharmacogenomics J..

[74] O’Donnell PH, Trubetskoy V, Nurhussein-Patterson A, Hall JP, Nath A, Huo D (2020). Clinical evaluation of germline polymorphisms associated with capecitabine toxicity in breast cancer: TBCRC-015. Breast Cancer Res Treat.

[75] Zhang X, Bai Z, Chen B, Feng J, Yan F, Jiang Z, et al. Polymorphisms of dihydropyrimidine dehydrogenase gene and clinical outcomes of gastric cancer patients treated with fluorouracil-based adjuvant chemotherapy in Chinese population. Chin Med J (Engl). 2012;125(5):741–6. 22490566

[76] Deenen MJ, Tol J, Burylo AM, Doodeman VD, de Boer A, Vincent A, Guchelaar HJ, Smits PHM, Beijnen JH, Punt CJA, Schellens JHM, Cats A (2011). Relationship between single nucleotide polymorphisms and haplotypes in DPYD and toxicity and efficacy of capecitabine in advanced colorectal cancer. Clin Cancer Res.

[77] Rosmarin D, Palles C, Church D, Domingo E, Jones A, Johnstone E, Wang H, Love S, Julier P, Scudder C, Nicholson G, Gonzalez-Neira A, Martin M, Sargent D, Green E, McLeod H, Zanger UM, Schwab M, Braun M, Seymour M, Thompson L, Lacas B, Boige V, Ribelles N, Afzal S, Enghusen H, Jensen SA, Etienne-Grimaldi MC, Milano G, Wadelius M, Glimelius B, Garmo H, Gusella M, Lecomte T, Laurent-Puig P, Martinez-Balibrea E, Sharma R, Garcia-Foncillas J, Kleibl Z, Morel A, Pignon JP, Midgley R, Kerr D, Tomlinson I (2014). Genetic markers of toxicity from capecitabine and other fluorouracil-based regimens: investigation in the QUASAR2 study, systematic review, and meta-analysis. J Clin Oncol Off J Am Soc Clin Oncol.

[78] McLeod HL, Sargent DJ, Marsh S, Green EM, King CR, Fuchs CS (2010). Pharmacogenetic predictors of adverse events and response to chemotherapy in metastatic colorectal cancer: results from north American gastrointestinal intergroup trial N9741. J Clin Oncol Off J Am Soc Clin Oncol.

[79] Goekkurt E, Al-Batran S-E, Hartmann JT, Mogck U, Schuch G, Kramer M (2009). Pharmacogenetic analyses of a phase III trial in metastatic gastroesophageal adenocarcinoma with fluorouracil and leucovorin plus either oxaliplatin or cisplatin: a study of the arbeitsgemeinschaft internistische onkologie. J Clin Oncol Off J Am Soc Clin Oncol.

[80] Kim HS, Kim M-K, Chung HH, Kim JW, Park NH, Song YS, Kang SB (2009). Genetic polymorphisms affecting clinical outcomes in epithelial ovarian cancer patients treated with taxanes and platinum compounds: a Korean population-based study. Gynecol Oncol.

[81] Booten R, Ward T, Heighway J, Ashcroft L, Morris J, Thatcher N (2006). Glutathione-S-Transferase P1 Isoenzyme polymorphisms, platinum-based chemotherapy, and non-small cell lung Cancer. J Thorac Oncol.

[82] Lecomte T, Landi B, Beaune P, Laurent-Puig P, Loriot M-A (2006). Glutathione S-transferase P1 polymorphism (Ile105Val) predicts cumulative neuropathy in patients receiving oxaliplatin-based chemotherapy. Clin Cancer Res.

[83] Pastorelli R, Cerri A, Mezzetti M, Consonni E, Airoldi L (2002). Effect of DNA repair gene polymorphisms on BPDE-DNA adducts in human lymphocytes. Int J Cancer.

[84] Benhamou S, Sarasin A (2002). ERCC2/XPD gene polymorphisms and cancer risk. Mutagenesis..

[85] Boige V, Mendiboure J, Pignon J-P, Loriot M-A, Castaing M, Barrois M, Malka D, Trégouët DA, Bouché O, le Corre D, Miran I, Mulot C, Ducreux M, Beaune P, Laurent-Puig P (2010). Pharmacogenetic assessment of toxicity and outcome in patients with metastatic colorectal Cancer treated with LV5FU2, FOLFOX, and FOLFIRI: FFCD 2000-05. J Clin Oncol.

[86] Haenisch S, May K, Wegner D, Caliebe A, Cascorbi I, Siegmund W (2008). Influence of genetic polymorphisms on intestinal expression and rifampicin-type induction of ABCC2 and on bioavailability of talinolol. Pharmacogenet Genomics.

[87] Taniguchi K, Wada M, Kohno K, Nakamura T, Kawabe T, Kawakami M, Kagotani K, Okumura K, Akiyama S, Kuwano M (1996). A human canalicular multispecific organic anion transporter (cMOAT) gene is overexpressed in cisplatin-resistant human cancer cell lines with decreased drug accumulation. Cancer Res.

[88] Cecchin E, D’Andrea M, Lonardi S, Zanusso C, Pella N, Errante D (2013). A prospective validation pharmacogenomic study in the adjuvant setting of colorectal cancer patients treated with the 5-fluorouracil/leucovorin/oxaliplatin (FOLFOX4) regimen. Pharmacogenomics J..

[89] Han B, Gao G, Wu W, Gao Z, Zhao X, Li L, Qiao R, Chen H, Wei Q, Wu J, Lu D (2011). Association of ABCC2 polymorphisms with platinum-based chemotherapy response and severe toxicity in non-small cell lung cancer patients. Lung Cancer.

[90] Cui J-J, Wang L-Y, Zhu T, Gong W-J, Zhou H-H, Liu Z-Q, Yin JY (2017). Gene-gene and gene-environment interactions influence platinum-based chemotherapy response and toxicity in non-small cell lung cancer patients. Sci Rep.

[91] Nichetti F, Falvella FS, Miceli R, Cheli S, Gaetano R, Fucà G, Infante G, Martinetti A, Antoniotti C, Falcone A, di Bartolomeo M, Cremolini C, de Braud F, Pietrantonio F (2019). Is a pharmacogenomic panel useful to estimate the risk of oxaliplatin-related neurotoxicity in colorectal cancer patients?. Pharmacogenomics J.

[92] Varma KA, Jayanthi M, Dubashi B, Shewade DG (2019). Influence of DPYD*9A, DPYD*6 and GSTP1 ile105val genetic polymorphisms on Capecitabine and Oxaliplatin (CAPOX) associated toxicities in colorectal Cancer (CRC) patients. Asian Pac J Cancer Prev.

[93] Khushman M, Patel GK, Hosein PJ, Laurini JA, Cameron D, Clarkson DR (2018). Germline pharmacogenomics of DPYD*9A (c.85T>C) variant in patients with gastrointestinal malignancies treated with fluoropyrimidines. J Gastrointest Oncol.

[94] Varma A, Jayanthi M, Dubashi B, Shewade DG, Sundaram R. Genetic influence of DPYD*9A polymorphism on plasma levels of 5-fluorouracil and subsequent toxicity after oral administration of capecitabine in colorectal cancer patients of south Indian origin. Drug Metab Pers Ther. 2020;0(0). 10.1515/dmpt-2020-0133. 10.1515/dmpt-2020-0133 32966231

[95] Botticelli A, Onesti CE, Strigari L, Occhipinti M, Di Pietro FR, Cerbelli B (2017). A nomogram to predict 5-fluorouracil toxicity: when pharmacogenomics meets the patient. Anti-Cancer Drugs.

[96] Russano M, Napolitano A, Ribelli G, Iuliani M, Simonetti S, Citarella F, Pantano F, Dell’Aquila E, Anesi C, Silvestris N, Argentiero A, Solimando AG, Vincenzi B, Tonini G, Santini D (2020). Liquid biopsy and tumor heterogeneity in metastatic solid tumors: the potentiality of blood samples. J Exp Clin Cancer Res.

[97] Gong W, Peng J, Yin J, Li X, Zheng W, Xiao L, Tan LM, Xiao D, Chen YX, Li X, Zhou HH, Liu ZQ (2017). Association between well-characterized lung cancer lncRNA polymorphisms and platinum-based chemotherapy toxicity in Chinese patients with lung cancer. Acta Pharmacol Sin.

[98] Leone P, Buonavoglia A, Fasano R, Solimando AG, De Re V, Cicco S, et al. Insights into the regulation of tumor angiogenesis by Micro-RNAs. J Clin Med. 2019;8(12). 10.3390/jcm8122030. 10.3390/jcm8122030 PMC6947031 31757094

[99] Powell NR, Zhao H, Ipe J, Liu Y, Skaar TC. Mapping the miRNA-mRNA Interactome in Human Hepatocytes and Identification of Functional mirSNPs in Pharmacogenes. Clin Pharmacol Ther. 2021;n/a n/a; 10.1002/cpt.2379. 10.1002/cpt.2379 PMC9007393 34314509

