# Supplementary material for: A case-control study of a combination of single nucleotide polymorphisms and clinical parameters to predict clinically relevant toxicity associated with fluoropyrimidine and platinum-based chemotherapy in gastric cancer
Source: BMC Cancer. 2021 Sep 16;21:1030. doi: 10.1186/s12885-021-08745-0 (PMC8444616; doi:10.1186/s12885-021-08745-0)
Supplement: Supplementary file 1 — Additional file 1: Supplementary Material S1.Supplementary Fig. S1. Overall survival rates in the study cohort, Supplementary Fig. S2. Calibration plot for the prognostic model, Supplementary Table S1. Demographic and clinic-pathological characteristics of study population (N = 93), Supplementary Table S2. Platinum plus fluoropyrimidine-based chemotherapy combined treatments used in gastric cancer patients (N = 93), Supplementary Table S3. Grades of toxicity in gastric cancer patients by the Common Toxicity Criteria for Adverse Events (CTCAE) 4.0, Supplementary Table S4. Sex subgroup association analysis of the SNPs DPYD (rs1801265). Supplementary Table S5. Models for hematological grade ≥ 3 toxicity in gastric cancer patients treated with platinum/fluoropyridines -based chemotherapy using multivariate analysis. Supplementary Table S6. Models for gastrointestinal grade ≥ 3 toxicity in gastric cancer patients treated with platinum/fluoropyridines -based chemotherapy using multivariate analysis. Supplementary Table S7. Models for neurological grade ≥ 3 toxicity in gastric cancer patients treated with platinum/fluoropyridines -based chemotherapy using multivariate analysis. Supplementary Table S8. Genotypic and allelic frequencies for the analyzed polymorphisms, Supplementary Table S9. ID assay for each of the analyzed polymorphisms. Supplementary Table S10. SNPs selection based in score for fluoropyrimidines. Supplementary Table S11. Final score for fluoropyrimidines. Supplementary Table S12. SNPs selection based in score for platinums. Supplementary Table S13. Final score for platinums. Supplementary Table S14. Sensitivity, specificity and accuracy calculations from a 2 × 2 confusion matrix. Supplementary Methods: Details of chemotherapy schemes, SNPs selection and classifications algorithm used. Supplementary Data File 1. All raw data used in this study. [file 12885_2021_8745_MOESM1_ESM.zip › Supplementary_Material_S1.docx]

# Supplementary Figures and Tables

## 1. Supplementary Figures

(A)


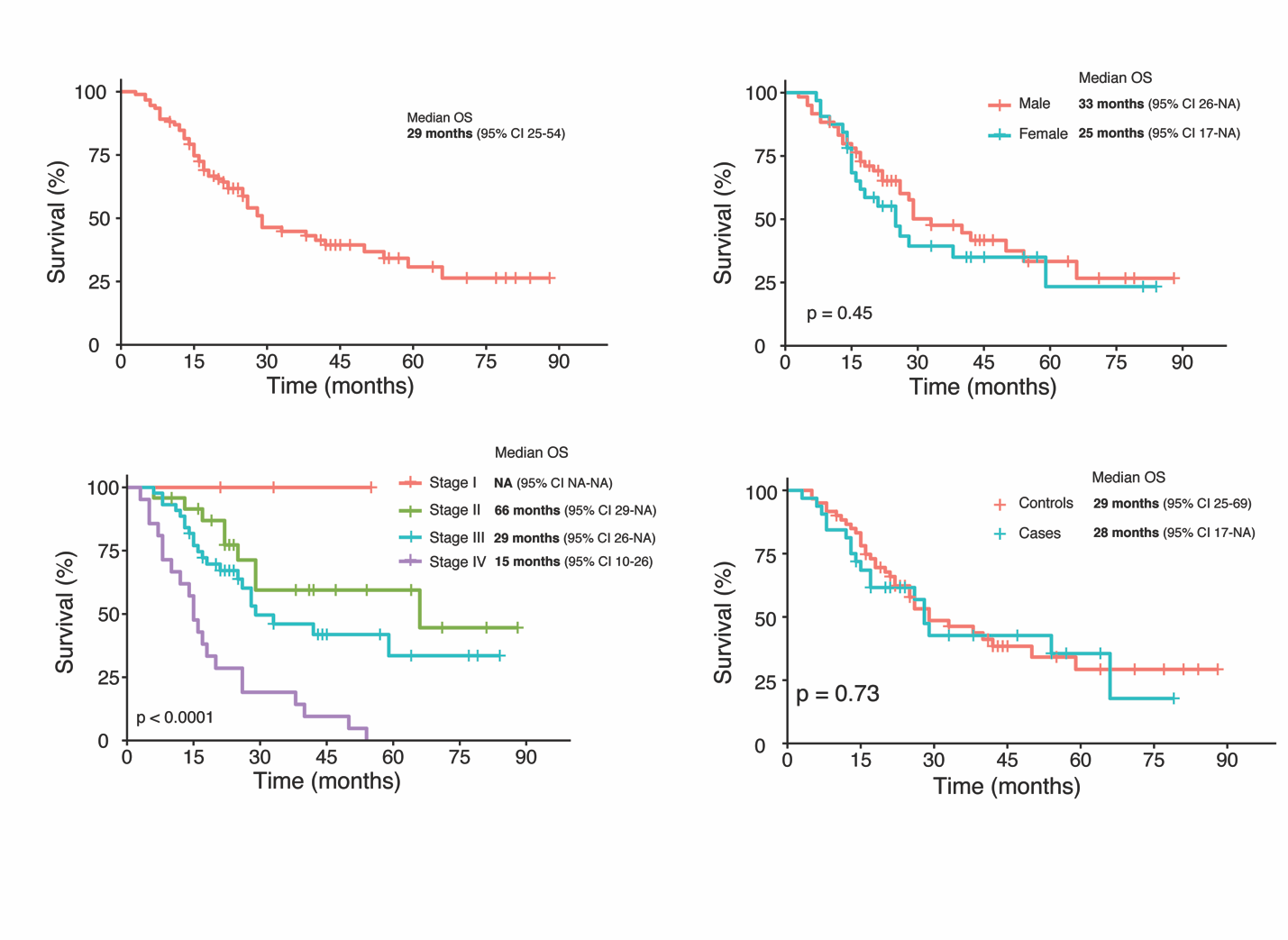


(C)

(D)

(B)

**Supplementary Figure S1**. Overall survival rates in the study cohort. Kaplan–Meier curves indicate overall survival for (A) the entire cohort, (B) by sex, (C) by cancer stage, and (D) by cases/controls. *Significance: p < 0.05*


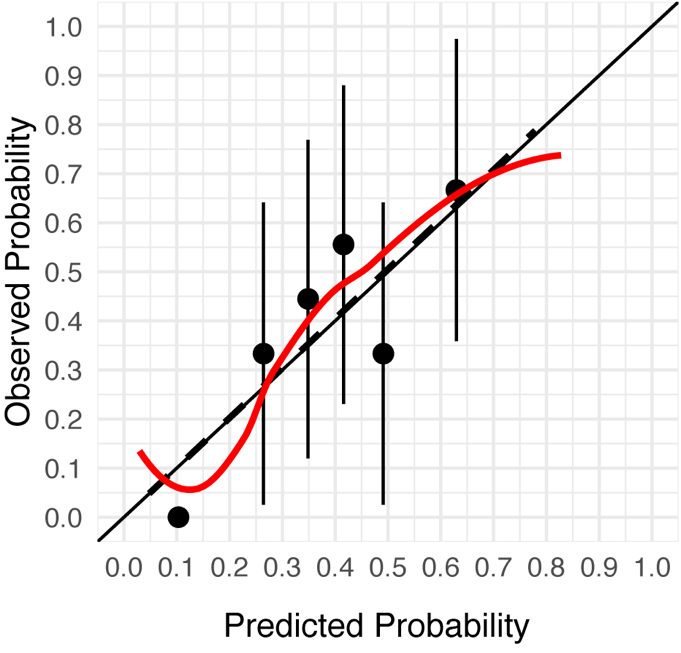


**Supplementary Figure S2.** Calibration plot for the prognostic model. Continuous line, ideal model; Red line, ideal model deviation, vertical bars; 95% confidence interval

| Characteristics | n (%) |
| --- | --- |
| Median age in years (range) | 59 (26-79) |
| Sex |  |
| Male | 58 (62.4) |
| Female | 33 (37.4) |
| Clinical stage |  |
| I | 3 (3.2) |
| II | 24 (25.8) |
| III | 44 (47.3) |
| IV | 22 (23.7) |
| ECOG |  |
| 0 | 42 (45.2) |
| 1 | 37 (39.8) |
| 2 | 3 (3.2) |
| ≥ 3 | 0 |
| NA | 11 (11.8) |
| Location primary tumor |  |
| Cardia | 11 (11.8) |
| Distal esophagus and GEJ | 14 (15.1) |
| Fundus | 5 (5.4) |
| Corpus | 29 (31.2) |
| Antrum | 19 (20.4) |
| Pylorus | 4 (4.3) |
| Multiple | 8 (8.6) |
| NA | 3 (3.2) |
| Lauren histological type |  |
| Intestinal | 26 (28.0) |
| Diffuse | 30 (32.3) |
| Mixed | 11 (11.8) |
| NA | 26 (28.0) |
| WHO histological type |  |
| Adenocarcinoma | 71 (76.3) |
| Undifferentiated carcinoma | 5 (5.4) |
| Adenosquamous cell carcinoma | 1 (1.1) |
| NA | 16 (17.2) |
| Signet-ring cell presence |  |
| No | 54 (58.1) |
| Yes | 39 (41.9) |

**Supplementary Table S1**. Demographic and clinic-pathological characteristics of study population (N = 93)

*GEJ, gastroesophageal junction; ECOG, Eastern Cooperative*

*Oncology Group; WHO; World Health Organization. NA. not available.*

**Supplementary Table S2**. Platinum plus fluoropyrimidine-based chemotherapy combined treatments used in gastric cancer patients (N = 93).

| Characteristic | n (%) |
| --- | --- |
| Chemotherapy regimen |  |
| Peri-operative | 32 (34.4) |
| Palliative | 30 (32.2) |
| Adjuvant | 26 (28.0) |
| CMT + RDT Adjuvant | 4 (4.3) |
| CMT + RDT Peri-operative | 1 (1.1) |
| Chemotherapy scheme |  |
| FOLFOX | 46 (49.5) |
| CAPEOX | 17 (18.3) |
| CF | 13 (14.0) |
| DCFm | 5 (5.4) |
| ECF | 2 (2.2) |
| EOX | 2 (2.2) |
| Capecitabine | 2 (2.2) |
| FLOT | 1 (1.1) |
| RDT + 5-FU/Leu | 3 (3.2) |
| RDT + CAPEOX | 1 (1.1) |
| RDT + Cis/Cape | 1 (1.1) |
| Previously treated |  |
| No | 83 (89.3) |
| Yes | 10 (10.7) |
| Scheme contains |  |
| RDT | 5 (5.3) |
| 5-FU | 70 (75.3) |
| Capecitabine | 21 (22.6) |
| Oxaliplatin | 67 (72.0) |
| Cisplatin | 21 (21.3) |
| Docetaxel | 6 (6.5) |
| Surgery |  |
| No | 10 (10.7) |
| Yes | 83 (89.3) |

*CMT. chemotherapy; RDT. Radiotherapy; FOLFOX. 5-fluorouracil+oxaliplatin+leucovorin; CAPEOX. capecitabine + oxaliplatin; CF. cisplatin + 5-fluorouracil; DCFm. docetaxel + cisplatin + 5-fluorouracil; ECF. etoposide + cisplatin + 5-fluorouracilo; FLOT. 5-fluorouracil + oxaliplatin + docetaxel + leucovorin; 5FU. 5-fluorouracil; Leu. leucovorin; Cis. cisplatin; Cape. capecitabine.*

| **Supplementary Table S3.** Grades of toxicity in gastric cancer patients by the Common Toxicity Criteria for Adverse Events (CTCAE) 4.0 | | | | | |
| --- | --- | --- | --- | --- | --- |
| **Toxicity** | ***n (%)*** | | | | |
|  | **Grade 0** | **Grade 1** | **Grade 2** | **Grade 3** | **Grade 4** |
| **Hematological** |  |  |  |  |  |
| Anemia | 87 (93.5) | 2 (2.2) | 3 (3.3) | 1 (1.1) | 0 |
| Neutropenia | 77 (84.7) | 1 (1.1) | 4 (4.4) | 8 (8.8) | 3 (3.3) |
| Febrile neutropenia | 89 (95.6) | 0 | 0 | 4 (4.4) | 0 |
| Thrombocytopenia | 82 (87.9) | 3 (3.3) | 7 (7.7) | 1 (1.1) | 0 |
| **Gastrointestinal** |  |  |  |  |  |
| Nausea | 56 (59.3) | 17 (18.7) | 17 (18.7) | 2 (2.2) | 1 (1.1) |
| Vomiting | 78 (83.5) | 10 (11.0) | 2 (2.2) | 3 (3.3) | 0 |
| Diarrhea | 68 (72.5) | 14 (15.4) | 4 (4.4) | 7 (7.7) | 0 |
| Stomatitis | 78 (83.5) | 6 (6.6) | 3 (3.3) | 5 (5.5) | 1 (1.1) |
| **Neurological** |  |  |  |  |  |
| Palmoplantar erythrodysesthesia | 85 (91.2) | 3 (3.3) | 3 (3.3) | 2 (2.2) | 0 |
| Peripheral neuropathy | 53 (56.0) | 27 (29.7) | 11 (12.1) | 2 (2.2) | 0 |
| * *No patient showed toxicity grade 5* | | | | | |

**Supplementary Table S4.** Sex subgroup association analysis of the SNPs *DPYD* (rs1801265).

**Males**

**Females**

| Gen SNP ID | Model | Genotypes | Control (n = 40)  n (%) | Case (n = 19)  n (%) | OR [95% CI] | p-value |
| --- | --- | --- | --- | --- | --- | --- |
| *DPYD* |  |  |  |  |  |  |
| rs1801265 A>G | |  |  |  |  |  |
|  | Dom | A/A | 27 (67.5%) | 4 (21.1%) | Ref |  |
|  |  | A/G-G/G | 13 (32.5%) | 15 (78.9%) | **7.78 (2.31-31.79)** | **0.001** |

| Gen SNP ID | Model | Genotypes | Control (n = 20)  n (%) | Case (n = 12)  n (%) | OR [95% CI] | p-value |
| --- | --- | --- | --- | --- | --- | --- |
| *DPYD* |  |  |  |  |  |  |
| rs1801265 A>G | |  |  |  |  |  |
|  | Dom | A/A | 13 (65.0%) | 6 (50.0%) | Ref |  |
|  |  | A/G-G/G | 7 (35.0%) | 6 (50.0%) | **1.85 (** **0.43-** **8.26)** | **0.40** |

*Dom: Dominant inheritance model.*

*Significance: p < 0.05*

|  | **Model 1** |  | **Model 2** |  | **Model 3** |  | **Model 4** |  |
| --- | --- | --- | --- | --- | --- | --- | --- | --- |
| **Characteristics** | **OR [95% CI]** | **p-value** | **OR [95% CI]** | **p-value** | **OR [95% CI]** | **p-value** | **OR [95% CI]** | **p-value** |
| Age (above median) | 1.82  (0.53-6.90) | 0.34 | - | - | 2.70  (0.65-13.71) | 0.19 | 3.57  (0.77-22.33) | 0.12 |
| Sex (Female) | 2.07  (0.62-6.98) | 0.22 | - | - | 3.39  (0.80-15.95) | 0.10 | **4.95**  (1.06-28.79) | **0.05** |
| Chronic liver disease (Yes) | 4.48  (0.15 -126.7) | 0.31 |  |  | 3.63  (0.11-129.5) | 0.42 | 11.61  (0.25-1029) | 0.20 |
| CMT Adjuvant (Yes) | 0.48  (0.06-2.43) | 0.41 | - | - | 0.53  (0.06-3.33) | 0.51 | 0.52 (0.05-3.46) | 0.52 |
| 5-FU based (Yes) | 1.57  (0.32-11.8) | 0.60 | - | - | 1.58  (0.27-13.41) | 0.62 | 1.44 (0.24-13.03) | 0.70 |
| **SNPs** |  |  |  |  |  |  |  |  |
| GSTP1 (dom. AG/GG) (rs1695) | - | - | 0.37  (0.10-1.24) | 0.11 | 0.25  (0.05-0.97) | 0.05 | - | - |
| DPYD (dom. AG/GG) (rs1801265) | - | - | 2.55  (0.76-9.35) | 0.13 | **3.19**  (0.87-13.16) | **0.08** | - | - |
| ERCC1 (rec. GG) (rs11615) | - | - | **3.35**  (0.84-12.71) | **0.07** | 3.12  (0.69-13.55) | 0.13 | - | - |
| **SNPs combination** |  |  |  |  |  |  |  |  |
| *ERCC1 rec.* + *GSTP1 dom. (rs11615 + rs1695)* | - | - | - | - | - | - | - | - |
| AA/AG + AG/GG | - | - | - | - | - | - | 0.58  (0.11-3.14) | 0.51 |
| GG + AA | - | - | - | - | - | - | **17.84**  (2.05-220.4) | **0.01** |
| GG + AG/GG | - | - | - | - | - | - | 0.25  (0.008-3.05) | 0.33 |
| DPYD (dom. AG/GG) (rs1801265) | - | - | - | - | - | - | **3.50**  (0.86-17.08) | **0.09** |
|  | **Model 1** |  | **Model 2** |  | **Model 3** |  | **Model 4** |  |
| **Pseudo R^2^** | 0.07 |  | 0.14 |  | 0.23 |  | 0.29 |  |

## Supplementary Table S5. Models for hematological grade ≥ 3 toxicity in gastric cancer patients treated with platinum/fluoropyridines -based chemotherapy using multivariate analysis.

*CMT. Chemotherapy; Dom: Dominant inheritance model; Rec: Recessive inheritance model Significance: P< 0.05*

**Supplementary Table S6.** Models for gastrointestinal grade ≥ 3 toxicity in gastric cancer patients treated with platinum/fluoropyridines -based chemotherapy using multivariate analysis.

|  | **Model 1** |  | **Model 2** |  | **Model 3** |  | **Model 4** |  |
| --- | --- | --- | --- | --- | --- | --- | --- | --- |
| **Characteristics** | **OR [95% CI]** | **p-value** | **OR [95% CI]** | **p-value** | **OR [95% CI]** | **p-value** | **OR [95% CI]** | **p-value** |
| Age (above median) | 1.54  (0.52-4.82) | 0.43 | - | - | 2.09  (0.61-8.11) | 0.25 | 1.53  (0.47-5.29) | 0.48 |
| Sex (Female) | 1.84  (0.56-6.04) | 0.30 | - | - | 1.68  (0.43-6.48) | 0.44 | 2.30  (0.64-8.66) | 0.20 |
| Cisplatin based (Yes) | 2.73  (0.78-9.43) | 0.10 | - | - | **3.63**  (0.79-17.11) | **0.09** | **4.30**  (1.06-18.7) | **0.04** |
| **SNPs** |  |  |  |  |  |  |  |  |
| *UMPS* (rec. CC) (rs1801019) | - | - | 4.30  (0.43-45.31) | 0.19 | 5.24  (0.43-71.88) | 0.18 | - | - |
| *DPYD* (dom. TT) (rs1801159) | - | - | **4.40**  (1.34-17.27) | **0.02** | **5.23**  (1.50-22.57) | **0.01** | - | - |
| *DPYD* (dom. AG/GG) (rs1801265) | - | - | 1.62  (0.50-5.47) | 0.41 | 1.91  (0.56-6.98) | 0.30 | - | - |
| **SNPs combination** |  |  |  |  |  |  |  |  |
| *ERCC1* rec*.* + *DPYD* dom.  (rs11615 + rs1801159) | - | - | - | - | - |  | - | - |
| AA/AG + CT/TT | - | - | - | - | - |  | **0.15**  (0.03-0.54) | **0.006** |
| GG + TT | - | - | - | - | - |  | NA | NA |
| GG + CT/TT | - | - | - | - | - |  | **0.13**  (0.05-1.01) | **0.09** |
|  | **Model 1** |  | **Model 2** |  | **Model 3** |  | **Model 4** |  |
| **Pseudo R^2^** | 0.04 |  | 0.15 |  | 0.20 |  | 0.18 |  |

*Dom: Dominant inheritance model; Rec: Recessive inheritance model*

*Significance: P< 0.05*

**Supplementary Table S7.** Models for neurological grade ≥ 3 toxicity in gastric cancer patients treated with platinum/fluoropyridines -based chemotherapy using multivariate analysis.

|  | **Model 1** |  | **Model 2** |  | **Model 3** |  |
| --- | --- | --- | --- | --- | --- | --- |
| **Characteristics** | **OR [95% CI]** | **p-value** | **OR [95% CI]** | **p-value** | **OR [95% CI]** | **p-value** |
| Age (above median) | 0.46 (0.02-5.43) | 0.55 | - | - | 0.62 (0.02-8.87) | 0.72 |
| Sex (Female) | 2.24 (0.23-22.60) | 0.45 | - | - | 2.12 (0.20-22.66) | 0.50 |
| ECOG (0 vs 1,2) | - | - | - | - | - | - |
| 1 | 2.69 (0.23-61.28) | 0.43 |  |  | 1.87 (0.14-44.70) | 0.63 |
| 2 | 35.76 (0.81-2337) | 0.06 |  |  | 31.37 (0.74-1910) | 0.06 |
| **SNPs** |  |  |  |  |  |  |
| TP53 (rec. GG) (rs1042522) | - | - | 5.46 (0.24-53.0) | 0.17 | 5.28 (0.19-80.47) | 0.24 |
|  | **Model 1** |  | **Model 2** |  | **Model 3** |  |
| **Pseudo R^2^** | 0.15 |  | 0.05 |  | 0.19 |  |

*Rec: Recessive inheritance model; ECOG: Eastern Cooperative Oncology Group*

*Significance: P< 0.05*

**Supplementary Table S8.** Genotypic and allelic frequencies for the analyzed polymorphisms.

| **Gen SNP ID** | **Frequencies n (%)** |
| --- | --- |
|  | **N = 93** |
| ***GSTP1* rs1695 A>G** |  |
| AA | 36 (39%) |
| AG | 44 (47%) |
| GG | 13 (14%) |
| Allele |  |
| A | 116 (62%) |
| G | 70 (38%) |
| ***ERCC2* rs13181 T>G** |  |
| TT | 55 (59%) |
| TG | 36 (39%) |
| GG | 2 (2%) |
| Allele |  |
| T | 146 (78%) |
| G | 40 (22%) |
| ***TP53* rs1042522 C>G** |  |
| CC | 56 (62%) |
| CG | 29 (32%) |
| GG | 6 (7%) |
| NA | 2 (2,15%) |
| Allele |  |
| C | 141 (77%) |
| G | 41 (23%) |
| ***UMPS* rs1801019 G>C** |  |
| GG | 61 (66%) |
| GC | 28 (30%) |
| CC | 4 (4%) |
| Allele |  |
| G | 150 (81%) |
| C | 36 (19%) |
| ***SHMT1* rs1979277 G>A** |  |
| GG | 50 (54%) |
| AG | 35 (38%) |
| AA | 7 (8%) |
| NA | 1 (1%) |
| Allele |  |
| G | 135 (73%) |
| A | 49 (27%) |
| ***MTHFR* rs1801131 T>G** |  |
| TT | 52 (56%) |
| TG | 36 (39%) |
| GG | 5 (5%) |
| Allele |  |
| T | 145 (75%) |
| G | 46 (25%) |
| ***ABCC2* rs717620 C>T** |  |
| CC | 76 (83%) |
| CT | 15 (16%) |
| TT | 1 (1%) |
| NA | 1 (1%) |
| Allele |  |
| C | 167 (91%) |
| T | 17 (9%) |
| ***DPYD* rs55886062 A>C** |  |
| AA | 92 (100%) |
| AC | 0 (0%) |
| CC | 0 (0%) |
| NA | 1 (1%) |
| Allele |  |
| A | 184 (100%) |
| C | 0 (0%) |
| ***DPYD* rs2297595 T>C** |  |
| TT | 85 (92%) |
| TC | 7 (8%) |
| CC | 0 (0%) |
| NA | 1 (1%) |
| Allele |  |
| T | 177 (96%) |
| C | 7 (4%) |
| ***DPYD* rs1801159 T>C** |  |
| TT | 40 (43%) |
| TC | 41 (45%) |
| CC | 11 (12%) |
| NA | 1 (1%) |
| Allele |  |
| T | 121 (66%) |
| C | 63 (34%) |
| ***DPYD* rs1801265 A>G** |  |
| AA | 50 (55%) |
| AG | 30 (33%) |
| GG | 11 (12%) |
| NA | 2 (2,15%) |
| Allele |  |
| A | 130 (71%) |
| G | 52 (29%) |
| ***ERCC1* rs11615 A>G** |  |
| AA | 16 (17%) |
| AG | 60 (65%) |
| GG | 16 (17%) |
| NA | 1 (1%) |
| Allele |  |
| A | 92 (50%) |
| G | 92 (50%) |

*NA, not available*

**Supplementary Table S9**. ID assay for each of the analyzed polymorphisms.

| **Gen SNP ID** | **ID assay** |
| --- | --- |
|  |  |
| *GSTP1* rs1695 A>G | C___3237198_20 |
| *ERCC2* rs13181 T>G | C___3145033_10 |
| *TP53* rs1042522 C>G | C___2403545_10 |
| *UMPS* rs1801019 G>C | C___1901477_10 |
| *SHMT1* rs1979277 G>A | C___3063127_10 |
| *MTHFR* rs1801131 T>G | C____850486_20 |
| *ABCC2* rs717620 C>T | C___2814642_10 |
| *DPYD* rs2297595 T>C | C__16187014_20 |
| *DPYD* rs1801159 T>C | C___1823316_20 |
| *DPYD* rs1801265 A>G | C___9491497_10 |
| *ERCC1* rs11615 A>G | C___2532959_20 |

# Supplementary Methods

1. Treatment schemes:

The treatment schemes for the group of patients were:

FOLFOX: Chemotherapy scheme that combines the 5-FU drug and oxaliplatin. In addition, leucovorin is added to increase the therapeutic efficacy of pyrimidine. Each cycle consists of 85 mg/m2 of oxaliplatin IV (day 1), 200 mg/m2/day of IV leucovorin (day 1 and 2), IV bolus of 400 mg/m2/ day of 5-FU (day 1 and 2) and continuous 24-hour IV infusion of 1200 mg/m2 of 5-FU (day 1). Each cycle is repeated every 15 days. Supportive care includes: Dexamethasone 12 mg IV, ondansetron 8 mg IV and famotidine 20 mg IV, all 30 minutes before begin chemotherapy.

CAPEOX: Chemotherapy scheme that combines the drugs capecitabine and oxaliplatin. Each cycle consists of 130 mg/m2 of oxaliplatin IV (day 1) and 2000 mg/m2/day of capecitabine P.O on day 1 through day 14. Each cycle is repeated every 21 days. Supportive care includes: Dexamethasone 12 mg IV, ondansetron 8 mg IV and famotidine 20 mg IV, all 30 minutes before begin chemotherapy.

CF: Chemotherapy scheme that combines the drugs cisplatin and 5-FU. Each cycle consists of 80 mg/m2 cisplatin IV (day 1) and 24-hour continuous IV infusion of 5-FU 800 mg/m2/day on day 1 through day 5. Each cycle is repeated every 21 days. Supportive care includes: Dexamethasone 12 mg IV, ondansetron 8 mg IV, famotidine 20 mg IV and aprepitant 125 mg P.O, all 30 minutes before begin chemotherapy.

DFCm: Chemotherapy scheme that combines the drugs docetaxel, cisplatin and 5-FU. Each cycle consists of 75 mg/m2 of docetaxel IV, 75 mg/m2 of cisplatin IV (day 1) and continuous 24-hour infusion of 750 mg/m2/day of 5-FU on day 1 through day 5. Each cycle repeated every 21 days. Supportive care includes: Dexamethasone 12 mg IV, ondansetron 8 mg IV, famotidine 20 mg IV, aprepitant 125 mg P.O and chlorphenamine 10 mg IV, all 30 minutes before begin chemotherapy.

ECF: Chemotherapy scheme that combines the drugs epirubicin, cisplatin and 5-FU. Each cycle consists of 50 mg/m2 epirubicin IV, 50 mg/m2 cisplatin IV (day 1), and 24-hour continuous IV infusion of 200 mg/m2/day 5-FU on day 1 repeated twice weekly until day 21. Each cycle is repeated every 21 days. Supportive care includes: Dexamethasone 12 mg IV, ondansetron 8 mg IV, famotidine 20 mg IV and aprepitant 125 mg P.O, all 30 minutes before begin chemotherapy.

EOX: Chemotherapy scheme that combines the drugs epirubicin, oxaliplatin and capecitabine. Each cycle consists of 130 mg/m2 of oxaliplatin IV (day 1), 50 mg/m2 of epirubicin IV (day 1) and 1250 mg/m2/day of capecitabine P.O on day 1 through day 14. Each cycle is repeated every 21 days. Dexamethasone 12 mg IV, ondansetron 8 mg IV and famotidine 20 mg IV, all 30 minutes before begin chemotherapy.

FLOT: Chemotherapy scheme that combines the drugs 5-FU, oxaliplatin, leucovorin and docetaxel. Each cycle consists of 85 mg/m2 of oxaliplatin IV (day 1), 200 mg/m2 of IV leucovorin (day 1), 50 mg/m2 of docetaxel IV (day 1) and continuous 24-hour IV infusion of 2600 mg/m2 of 5-FU (day 1). Each cycle is repeated every 15 days. Supportive care includes: Dexamethasone 12 mg IV, ondansetron 8 mg IV and famotidine 20 mg IV, all 30 minutes before begin chemotherapy.

Capecitabine: Monotherapy. Each cycle consists of 2000 mg/m2/ day of capecitabine P.O on day 1 through day 14. Each cycle is repeated every 21 days.

2. SNPs selection

The candidate SNPs for this association study were selected based on a scoring system according to the following criteria. The first criterion (1) used was the scientific evidence regarding the genetic variant toxicity relation, using the PharmGKB database. Where level 1 evidence means that the associations have been verified in different studies and level 4 means that there is only in-vitro or case report-based evidence. For level of evidence 1 a score of 4 is assigned, level 2 a score of 3, level 3 a score of 2 and for level 4 a score of 1. The second (2) criterion was the frequency of the risk allele of the corresponding SNPs according to the 1000-G Project database, considering as reference the data of the American population at a frequency of the allele associated with risk from 1% to 25%, from 26% to 50%, from 51% to 75% and from 76 to 99%, a value of 1,2,3, and 4 was assigned, respectively. The third (3) criterion is based on the relationship that the SNP has with the most clinically relevant adverse effect. For this study, the most relevant toxicities are hematological, neurological, and gastrointestinal. If the association corresponds to the most relevant adverse effect, it assigns a value of 2 and if it does not correspond it assigns a value of 1. The fourth (4) criterion used is in relation to the functional effect of the SNP on the corresponding protein. For a change in the inter/ intragenic, intronic or silent changes a value of 2 is assigned and for a change in the amino acid sequence a value of 4. Finally, an extra score was added to the SNPs that could alter the function of the protein. This from the use of prediction programs, these programs were PolyPhen and SIFT. The first is based on the conservation of sequences, folding and the crystal structure of the protein, instead, the second program uses sequence homologies to try to predict the molecular consequences of the replacement of an amino acid. According to the Polyphen (5) score, a value of 0.5 was assigned for benign variations and 1 for deleterious ones. According to the score obtained in SIFT(6), for the tolerated variations a score of 0.5 was assigned and for the damaging ones of 1. For the final selection, the scores obtained by each SNP in fluoropyrimidines and platins are averaged, the choice was from those SNPs that had a score equal to or greater than the average. Detailed analysis of the score for fluoropyrimidines is found in **Supplementary Table S10 and Supplementary Table S11**, and for the platinums in **Supplementary Table S12 and Supplementary Table S13**. For fluoropyrimidines, 14 SNPs in 7 different genes were candidates (**Supplementary Table S11**of this section, those selected were marked in blue), while for platinums, 4 SNPs in 4 different genes were candidates (**Supplementary Table S13** of this section, those selected were marked in blue). Finally, eleven SNPs with an allelic frequency greater than 5% were genotyped (See Manuscript).

**Supplementary Table S10**. SNPs selection based in score for fluoropyrimidines.

| **Gen** | **SNP id** | **(1)** | **(2)** | **(3)** | **(4)** | **(5)** | **(6)** |
| --- | --- | --- | --- | --- | --- | --- | --- |
| *ABCC11* | rs7194667 | **3** | **G (0.06)** | Hema. toxicity | Intronic | NA | NA |
| *ABCC2* | rs717620 | **3** | **T (0.17)** | Overall toxicity | Intragenic | NA | NA |
| *ABCC4* | rs9561778 | **3** | **T (NA)** | Overall toxicity | Intronic | NA | NA |
| *ABCC5* | rs3749438 | **3** | **A (0.29)** | Overall toxicity | Intronic | NA | NA |
| *ABCC5* | rs10937158 | **3** | **C (0.59)** | Overall toxicity | Intronic | NA | NA |
| *ABCC5* | rs1045642 | **3** | **A (0.43)** | Overall toxicity | Silent | NA | NA |
| *ABCG1* | rs225440 | **3** | **T ((0.42)** | Hema. toxicity | Intronic | NA | NA |
| *DPYD* | rs67376798 | **1A** | **A (0.00)** | G.I Toxicity | Aa change | 0 | 0.61 |
| *DPYD* | rs3918290 | **1A** | **T (0.00)** | Overall toxicity | Intronic | NA | NA |
| *DPYD* | rs55886062 | **1A** | **C (0.00)** | Overall toxicity | Aa change | 0 | 0.977 |
| *DPYD* | rs2297595 | **2A** | **C (0.06)** | Overall toxicity | Aa change | 0 | 0.976 |
| *DPYD* | rs1801159 | **3** | **C (0.27)** | Overall toxicity | Aa change | 0.04 | 0.001 |
| *DPYD* | rs1801158 | **3** | **T (0.01)** | Overall toxicity | Aa change | 0.01 | 0.767 |
| *DPYD* | rs1801265 | **3** | **G (0.22)** | Overall toxicity | Aa change | 0.41 | 0 |
| *DPYD* | rs17376848 | **3** | **G (0.08)** | Overall toxicity | Silent | NA | NA |
| *DPYD* | rs75017182 | **3** | **G (0.99)** | Overall toxicity | Intronic | NA | NA |
| *DPYD* | rs115232898 | **3** | **C (0.00)** | Overall toxicity | Aa change | 0.01 | 0.95 |
| *ERCC1* | rs11615 | **2B** | **A (0.39)** | Nephrotoxicity | Silent | NA | NA |
| *ERCC2* | rs13181 | **3** | **T (0.79)** | Hema. and G.I toxicity | Trunc. | NA | NA |
| *GSTP1* | rs1695 | **2A** | **A (0.52)** | Cardiotoxicity | Aa change | 1 | 0.001 |
| *MTHFR* | rs1801131 | **3** | **T (0.15)** | Overall toxicity | Aa change | 0.15 | 0.005 |
| *PARD3B* | rs17626122 | **3** | **T (0.37)** | Overall toxicity | Intronic | NA | NA |
| *SHMT1* | rs1979277 | **3** | **A (0.27)** | Overall toxicity | Aa change | 0.04 | 0 |
| *TP53* | rs1042522 | **2B** | **G (0.32)** | Overall toxicity | Aa change | 0.57 | 0.143 |
| *TYMP* | rs11479 | **3** | **A (0.17)** | Overall toxicity | Aa change | Trunc. | Trunc. |
| *TYMS* | rs183205964 | **3** | **C (0.00)** | Overall toxicity | Intronic | NA | NA |
| *UMPS* | rs1801019 | **2B** | **C(0.26)** | G.I Toxicity | Aa change | 0.32 | 0.025 |

*NA; not applicable. Trunc; Truncated. Hema; Hematological. G.I, Gastrointestinal. Aa; Amino-acid.*

**Supplementary Table S11**. Final score for fluoropyrimidines.

| **Gen** | **SNP id** | **(1)** | **(2)** | **(3)** | **(4)** | **(5)** | **(6)** | **Final Score** |
| --- | --- | --- | --- | --- | --- | --- | --- | --- |
| GSTP1 | rs1695 | **3** | 3 | 2 | 4 | 0.5 | 0.5 | **13** |
| ERCC2 | rs13181 | **2** | 4 | 2 | 4 | 0 | 0 | **12** |
| TP53 | rs1042522 | **2** | 3 | 2 | 4 | 0.5 | 0.5 | **12** |
| UMPS | rs1801019 | **3** | 2 | 2 | 4 | 0.5 | 0.5 | **12** |
| DPYD | rs55886062 | **4** | 0 | 2 | 4 | 1 | 1 | **12** |
| DPYD | rs2297595 | **3** | 1 | 2 | 4 | 1 | 1 | **12** |
| SHMT1 | rs1979277 | **2** | 2 | 2 | 4 | 1 | 0.5 | **11.5** |
| DPYD | rs1801159 | **2** | 2 | 2 | 4 | 1 | 0.5 | **11.5** |
| DPYD | rs67376798 | **4** | 0 | 1 | 4 | 1 | 1 | **11** |
| DPYD | rs1801158 | **2** | 1 | 2 | 4 | 1 | 1 | **11** |
| DPYD | rs75017182 | **2** | 4 | 2 | 2 | 0 | 0 | **10** |
| MTHFR | rs1801131 | **2** | 1 | 2 | 4 | 0.5 | 0.5 | **10** |
| DPYD | rs1801265 | **2** | 1 | 2 | 4 | 0.5 | 0.5 | **10** |
| DPYD | rs115232898 | **2** | 0 | 2 | 4 | 1 | 1 | **10** |
| *ABCC5* | rs10937158 | **2** | 3 | 2 | 2 | 0 | 0 | **9** |
| *TYMP* | rs11479 | **2** | 1 | 2 | 4 | 0 | 0 | **9** |
| *ERCC1* | rs11615 | **3** | 2 | 2 | 2 | 0 | 0 | **9** |
| *ABCG1* | rs225440 | **2** | 2 | 2 | 2 | 0 | 0 | **8** |
| *ABCC5* | rs1045642 | **2** | 2 | 2 | 2 | 0 | 0 | **8** |
| *DPYD* | rs3918290 | **4** | 0 | 2 | 2 | 0 | 0 | **8** |
| *ABCC5* | rs3749438 | **2** | 2 | 2 | 2 | 0 | 0 | **8** |
| *PARD3B* | rs17626122 | **2** | 2 | 2 | 2 | 0 | 0 | **8** |
| *ABCC11* | rs7194667 | **2** | 1 | 2 | 2 | 0 | 0 | **7** |
| *ABCC2* | rs717620 | **2** | 1 | 2 | 2 | 0 | 0 | **7** |
| *DPYD* | rs17376848 | **2** | 1 | 2 | 2 | 0 | 0 | **7** |
| *ABCC4* | rs9561778 | **2** | 0 | 2 | 2 | 0 | 0 | **6** |
| *TYMS* | rs183205964 | **2** | 0 | 2 | 2 | 0 | 0 | **6** |
|  |  |  |  |  |  |  |  |  |
|  | **Average Score** | **10** |  |  |  |  |  |  |

**Supplementary Table S12**. SNPs selection based in score for platinums.

| **Gen** | **SNP id** | **(1)** | **(2)** | **(3)** | **(4)** | **(5)** | **(6)** |
| --- | --- | --- | --- | --- | --- | --- | --- |
| *XRCC1* | rs25487 | **2B** | **C (0.69)** | Hema. toxicity | Aa change | 0.6 | 0 |
| *ERCC1* | rs3212986 | **2B** | **C (0.65)** | Nefrotoxicidad | 3´ UTR | NA | NA |
| *PARD3B* | rs17626122 | **3** | **T (0.38)** | Hema. toxicity | Intronic | NA | NA |
| *ABCC2* | rs717620 | **3** | **C (0.83)** | Neurotoxicity | 5`UTR | NA | NA |
| *ABCC2* | rs717620 | **3** | **T (0.17)** | Hema. toxicity | 5`UTR | NA | NA |
| *GSTP1* | rs1695 | **3** | **A (0.52)** | Neurotoxicity | Aa change | 1 | 0.001 |
| *ERCC2* | rs13181 | **3** | **G (0.21)** | Hema. toxicity | Trunc. | NA | NA |
| *ERCC1* | rs11615 | **3** | **A (0.39)** | Hema. toxicity | Aa change | NA | NA |

*NA; not applicable. Trunc; Truncated. Hema; Hematological. G.I, Gastrointestinal. Aa; Amino-acid.*

**Supplementary Table S13**. Final score for platinums.

| **Gen** | **SNP id** | **(1)** | **(2)** | **(3)** | **(4)** | **(5)** | **(6)** | **Final Score** |
| --- | --- | --- | --- | --- | --- | --- | --- | --- |
| *XRCC1* | rs25487 | **3** | 3 | 2 | 4 | 0.5 | 0.5 | 13 |
| *GSTP1* | rs1695 | **2** | 3 | 2 | 4 | 0.5 | 0.5 | 12 |
| *ABCC2* | rs717620 | **2** | 4 | 2 | 2 | 0 | 0 | 10 |
| *ERCC1* | rs11615 | **2** | 2 | 2 | 4 | 0 | 0 | 10 |
| *ERCC1* | rs3212986 | **3** | 3 | 1 | 2 | 0 | 0 | 9 |
| *ERCC2* | rs13181 | **2** | 1 | 2 | 4 | 0 | 0 | 9 |
| *PARD3B* | rs17626122 | **2** | 2 | 2 | 2 | 0 | 0 | 8 |
| *ABCC2* | rs717620 | **2** | 1 | 2 | 2 | 0 | 0 | 7 |
|  |  |  |  |  |  |  |  |  |
|  | **Average Score** | **10** |  |  |  |  |  |  |

3. Classification algorithms

Seven commonly used classification algorithms were employed(1,2). The output consisted of severe toxicity (grade 0-2 vs grade 3-4), as a binary variable. For all algorithms, 5-fold cross-validation was used to evaluate the prediction of the models. In addition, 60% of the patients were assigned to the training group and 40% to the testing group. Briefly, the data was randomly and alternately divided into 5 groups, in each cross validation 4 groups were used to train the model and 1 group to test the prediction accuracy. This was repeated 5 times and the average was used as the overall prediction value. In addition, all the algorithms were performed with the default parameters. Therefore, without seeking the maximization of each one of them.

1. Logistic Regression (LR)

It is a multivariate method that models how a binary response variable depends on a set of explanatory variables.

It can be written as follows:

$$P (Y=1│X_{1},X_{2},\cdots,X_{n})=\frac{1}{1+e^{-(\beta_{0}+\beta_{1}x_{1}+\beta_{2}x_{2}\cdots+\beta_{n}x_{n})}}$$

Where, $P \left( Y=1 | X_{1},X_{2},\cdots,X_{n} \right)$

is the probability that a patient develops severe toxicity, $X_{1},X_{2},\cdots,X_{n}$are the predictors, $\beta_{0}$is the intercept and $\beta_{1},\beta_{2},\cdots,\beta_{n}$are the regression coefficients.

1. Support Vector Machine (SVM)

Support Vector Machine is a supervised machine learning model used to classification problems. We used linear SVM, which creates a linear functional to separate the variables according to the different classes (in our case, severe toxicity). The objective of the support vector machine algorithm is to find a hyperplane in an N-dimensional space (N - the number of features) that distinctly classifies the data points. For classification we used package "kernlab" with tuning parameters: Cost (C, numeric)(3).

1. Naïve Bayesian (NB)

This algorithm of classification is based on applying “Bayes theorem” with the “naive” assumption of conditional independence between every pair of features given the value of the class variable. Bayes' theorem can be written:

$$P (H│X_{1},X_{2},\cdots,X_{n})=\frac{P\left( X_{1},X_{2},\cdots,X_{n} | H \right)P\left( H \right)}{P\left( X_{1},X_{2},\cdots,X_{n} \right)}$$

Where, $P (H│X_{1},X_{2},\cdots,X_{n})$ is the posterior probability of a patient of having or not having severe toxicity, $X_{1},X_{2},\cdots,X_{n}$are the variables of each patient, P(H) is the prior probability that resultant from the training sample and P(X) is the marginal probability that the attribute appears in the training sample.

1. K-Nearest Neighbor (KNN)

This algorithm classifies the outcomes values by searching for the “most similar” data points by proximity learned in the training stage. To measure the proximity between variables, Euclidean distance is used. Accuracy was used to select the optimal K-value(3).

1. Artificial Neural Network (ANN)

Artificial Neural Network (ANN) is a network of groups of small processing units that are modeled based on the behavior of human neural networks. We used Feed-Forward Neural Network. The network is composed of different layers in which the nodes are distributed. The nodes in the “input” layers receive the information, then nodes in the “hidden” layers process them and these finally in the “output” layers deliver the classification. In our case input was variables of patients, while output was severe toxicity. Accuracy was used to select the optimal number of layers and decays(3).

1. Random Forest (RF)

Random Forest is a tree-based classification model. It used many classification or regression tree models to random subsets of the input data and uses the combined result (the forest) for prediction. It is a classification technique with a powerful performance because it uses bagging and random variable selection for tree building. Accuracy was used to select the number of variables available for splitting at each tree node (mtry), split rule and minimal node size(3).

1. Decision Tree (DT)

It is a classification technique with a powerful performance because it uses bagging and random variable selection for tree building. Accuracy was used to select the number of variables available for splitting at each tree node (mtry), split rule and minimal node size(3).

1. Performance evaluation

The performance of each model was estimated according to the calculation of sensitivity, specificity and accuracy. Using a 2x2 confusion matrix, each of the parameters is calculated as follows.

**Supplementary Table S14.** Sensitivity, specificity and accuracy calculations from a 2x2 confusion matrix.

|  | **Reference** |  |
| --- | --- | --- |
| **Predicted** | Event | No Event |
| Event | **A** | **B** |
| No event | **C** | **D** |

Sensitivity = A / (A + C)

Specificity = D / (B + D)

Accuracy = (A + D) / (A + B + C + D)

Also, the predictive capacity was evaluated by the area under curve (AUC) from the receiver operating characteristic (ROC) curve.

**References**

1. Han J, Kamber M, Pei J. 8 - Classification: Basic Concepts. In: Han J, Kamber M, Pei JBT-DM (Third E, editors. The Morgan Kaufmann Series in Data Management Systems [Internet]. Boston: Morgan Kaufmann; 2012. p. 327–91. Available from: http://www.sciencedirect.com/science/article/pii/B9780123814791000083

2. Yin J-Y, Li X, Li X-P, Xiao L, Zheng W, Chen J, et al. Prediction models for platinum-based chemotherapy response and toxicity in advanced NSCLC patients. Cancer Lett. 2016 Jul;377(1):65–73.

3. Kuhn M. Building Predictive Models in R Using the caret Package. J Stat Software; Vol 1, Issue 5 [Internet]. 2008; Available from: https://www.jstatsoft.org/v028/i05
